# Aerobic Exercise-Induced TGF-β Receptor Reprogramming Disrupts Neutrophil–Microglia Crosstalk to Attenuate Early Brain Injury after Subarachnoid Hemorrhage

**DOI:** 10.34133/research.1301

**Published:** 2026-05-28

**Authors:** Shengming Jiang, Li Jiang, Shiqiang Zhang, Xincan Zhao, Peipei Jiang, Qi Tian, Chengli Liu, Peibang He, Zhijie Li, Guijun Wang, Zhou Sun, Minghao Du, Zhongyang Zhang, Youyu Wang, Fuhai Chao, Yang Yuan, Yuwei Jiang, Zhan Zhang, Jianming Liao, Mingchang Li

**Affiliations:** ^1^ Department of Neurosurgery, Renmin Hospital of Wuhan University, Wuhan, Hubei 430060, China.; ^2^ Department of Neurology, The Affiliated Nanhua Hospital, Hengyang Medical School, University of South China, Hengyang, Hunan 421001, China.; ^3^ Department of Neurosurgery, Tongren Hospital Affiliated to Wuhan University (The Third Hospital of Wuhan), Wuhan, Hubei 430060, China.; ^4^ Central Laboratory, Renmin Hospital of Wuhan University, Wuhan, Hubei 430060, China.; ^5^ Department of Administration Office, The Affiliated Nanhua Hospital, Hengyang Medical School, University of South China, Hengyang, Hunan 421001, China.; ^6^ Department of Rehabilitation, Renmin Hospital of Wuhan University, Wuhan, Hubei 430060, China.; ^7^ State Key Laboratory of Metabolism and Regulation in Complex Organisms, Taikang Center for Life and Medical Sciences, Wuhan University, Wuhan, Hubei 430072, China.

## Abstract

Acute-phase neuroinflammation and early brain injury progressing rapidly is responsible for substantial severity and mortality in subarachnoid hemorrhage (SAH). While cohort studies have confirmed that aerobic exercise (AE) was associated with decreased short-term mortality, its precise mechanisms remain elusive. We employed a murine SAH model subjected to preconditioning AE to validate the above hypothesis, combining early neurological function assessment and histological staining. Whole-transcriptome profiling and single-cell RNA sequencing were performed to capture differentially expressed genes and to delineate subtype-specific differentiation. To recapitulate the neuroimmune crosstalk in vitro, we established a primary neutrophil–microglia coculture system. Targeted receptor–ligand interaction studies were employed to validate the specific environmental variables driving their synergistic activation. Our data indicated that AE significantly ameliorated neurological outcomes, cerebral edema, and neuronal apoptosis post-SAH. Sequencing data identified classical pro-inflammatory M1-like microglia, alongside a novel early-response microglial subset characterized by high expression of transforming growth factor beta 1; both populations were regulated by AE for modulating neuroinflammation and significantly correlated with infiltrating neutrophil counts. This crosstalk was mediated by mature neutrophil-derived leucine-rich alpha-2-glycoprotein 1 (Lrg1), which differentially activated the microglial transforming growth factor beta signaling pathway. Collectively, AE-mediated suppression of neutrophil infiltration rebalanced microglial differentiation, thereby attenuating neuroinflammation following SAH. We identified Lrg1 as a key immune mediator in this process, highlighting exercise-induced reprogramming at the receptor level as a potential therapeutic strategy for SAH.

## Introduction

Subarachnoid hemorrhage (SAH) represents a life-threatening neurological emergency characterized by disproportionately high morbidity and mortality, and long-term disability. Despite advances in microsurgical clipping and endovascular coiling to secure ruptured aneurysms, the neurological outcomes for patients have improved only modestly over recent decades [1,2]. The pathological progression and poor outcomes following SAH are largely determined by early brain injury (EBI), a process unfolding within the first 72 h that establishes the neuropathological substrate for subsequent disease advancement through an orchestrated cascade of molecular events, including acute neuronal apoptosis, blood–brain barrier (BBB) disruption, cerebral edema, and sustained neuroinflammatory responses [3].

Emerging evidence has established dysregulated neuroinflammation as a central driver of EBI progression. The initial hemorrhage triggers the release of damage-associated molecular patterns (DAMPs), which activate resident microglia toward a pro-inflammatory phenotype [4]. A dynamically reinforcing inflammatory loop between microglia and neutrophils has been known as a key mechanism worsening EBI after SAH [5]. Neutrophils, upon entering the central nervous system, release soluble factors including migration inhibitory factor-related protein 14 and integrin α9, as well as structured neutrophil extracellular traps (NETs), which collectively induce pyroptosis and inflammatory activation in microglia. These activated microglia escalate the response by secreting interleukin 1 beta (Il-1b), which further recruits and primes neutrophils, establishing a feed-forward cycle that fuels sustained neuroinflammation [6,7]. Clinically, this interplay is highly consequential: high levels of neutrophil-related inflammatory markers, the neutrophil-to-lymphocyte ratio and secreted phosphoprotein 1 (Spp1), robustly predict increased mortality and poor functional recovery [8,9]. Consequently, targeting this maladaptive crosstalk between infiltrating immune cells and resident glial populations has been proposed as a promising therapeutic strategy for SAH [10].

The recent surge of interest in exercise-mediated neuroprotection is catalyzed by seminal reports identifying blood-derived exercise factors clusterin and glycosylphosphatidylinositol-specific phospholipase D1, which confer broad neuroprotection by attenuating neuroinflammation and promoting neurogenesis in aging and neurodegeneration [11–13]. Prospective cohort studies have suggested that prestroke exercise is associated with improved functional recovery and is widely recognized as a key behavioral intervention for the primary and secondary prevention of cardiovascular and cerebrovascular disorders [14,15]. Specifically, appropriate prestroke exercise combined with light-to-moderate endurance training, such as aerobic exercise (AE), is considered safe and can decrease early mortality and alleviate acute declines in neurological function [16,17]. However, the specific cellular and molecular mechanisms by which AE preconditioning reconfigures the immune landscape to protect against SAH-induced EBI remain largely unexplored.

In the present study, we hypothesized that AE preconditioning alleviated neuroinflammation and subsequent EBI by disrupting the pathogenic crosstalk between infiltrating neutrophils and microglia. To test this, we established a murine SAH model with a preceding regimen of daily AE, combined histological analyses and unbiased transcriptomic profiling at both the bulk and single-cell level to clarify the immune status, and further employed in vitro coculture systems to validate the signaling mechanisms identified. Our data revealed that AE preconditioning significantly improved neurological outcomes and attenuated cerebral edema and neuronal apoptosis after SAH. Single-cell RNA sequencing (scRNA-seq) not only identified classical pro-inflammatory M1-like microglia but also uncovered a novel early-response microglia with high expression of transforming growth factor beta 1 (Tgfb1^high^). Notably, the abundance of both subsets was modulated by AE and strongly correlated with the degree of neutrophil infiltration. Mechanistically, we identified neutrophil-derived leucine-rich alpha-2-glycoprotein 1 (Lrg1) as a key mediator that differentially activated the microglial TGF-β signaling pathway—a process potently suppressed by prior AE. Collectively, these findings identified Lrg1 as a critical immune checkpoint and establish AE-induced receptor-level reprogramming as a novel therapeutic strategy for SAH, providing new insights into the neuroimmune mechanisms underlying AE preconditioning in hemorrhagic stroke.

## Results

### AE alleviates EBI and neuroinflammation of SAH

**Fig. 1.**
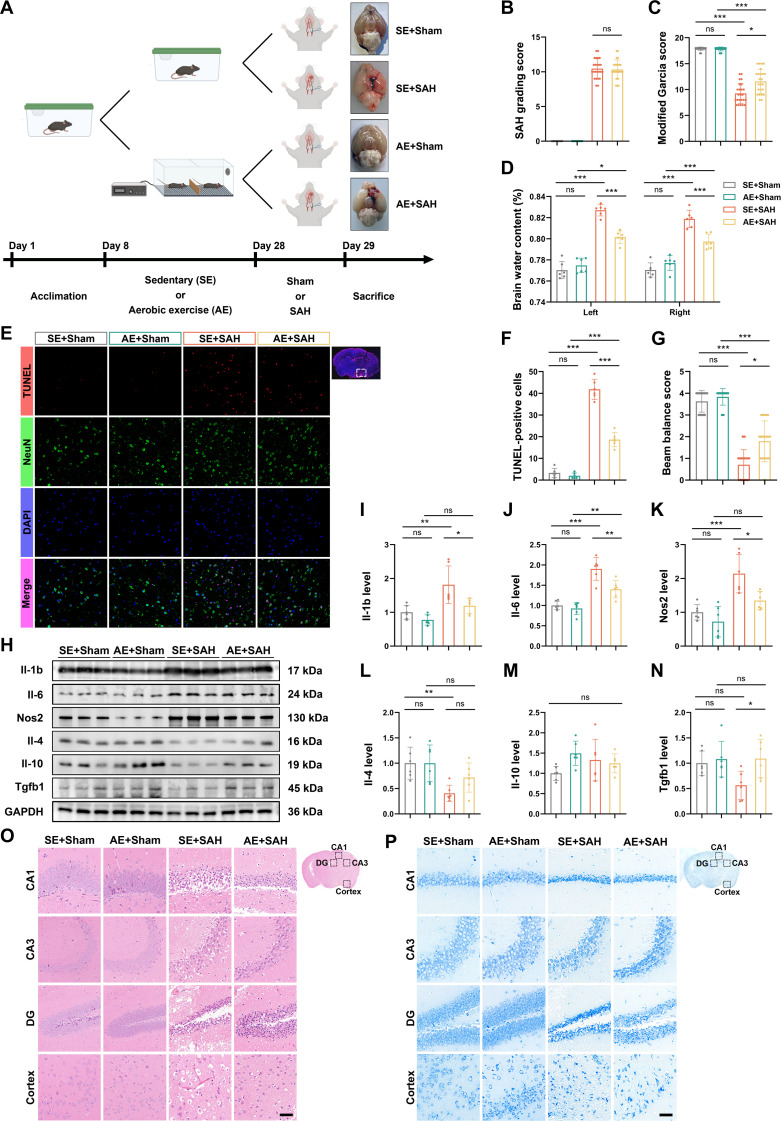
Animal study verifying the effect of AE in mitigating EBI of SAH. (A) The complete timeline of an experimental plan, schematic diagram, and actual skull base image of the SAH/Sham model in this animal experiment. Some elements are created in BioRender (2026); https://BioRender.com/vwwir98. (B) SAH grading score of each group in this stage. *n* = 24. (C) Modified Garcia score of each group, *n* = 24. (D) The water content of the left and right brains in different groups at this stage. *n* = 6. (E and F) Apoptosis of bleeding focus cortical neurons was detected by TUNEL assay (red), NeuN (green), and DAPI (blue) immunofluorescence. *n* = 6. Scale bars, 50 μm. (G) Bars show changes in BBs in SAH mice. *n* = 24. (H to N) Western blotting images and quantitative data of relative expression level of Il-1b, Il-6, Nos2, Il-4, Il-10, and Tgfb1 in the ipsilateral cortex after SAH. *n* = 6. (O) The coronal sections were stained with hematoxylin and eosin (H&E) for histological evaluation AE-regulated in SAH. *n* = 6. Scale bars, 50 μm. (P) Histopathological changes detected by Nissl’s staining in hippocampus and the hemorrhagic cortex. *n* = 6. Scale bars, 50 μm. In (B), (C), and (G), data were represented as median (interquartile range), and *P* values were calculated using Kruskal–Wallis *H* with Dunn test. In (D), (F), and (I) to (N), data were represented as mean ± SD, and *P* values were calculated using one-way ANOVA with Tukey multiple comparisons. *P < 0.05, **P < 0.01, ***P < 0.001. The original Western blot images are shown in Data S1. Detailed experimental data and statistical results, including *Z* and *P* values for Kruskal–Wallis test and *F* and *P* values for ANOVAs, are provided in Data S7 to S10.

Figure 1A outlines the Experiment 1 timeline and confirms successful SAH or sham modeling. Among the 59 mice subjected to SAH induction in Experiment 1 (total *n* = 112), 11 (18.64%) died post-procedure (Table S1), with no significant differences in SAH grading scores across SAH groups (Fig. 1B). Neurological assessments 24 h post-SAH revealed that both the Modified Garcia score (MGs) and beam balance score (BBs) were significantly impaired in the sedentary (SE)+SAH group compared to the SE+Sham group. Although the AE+SAH group also scored lower than the AE+Sham group, it exhibited significantly better performance than the SE+SAH group (Fig. 1C and G).

Brain edema analysis demonstrated markedly elevated water content in both hemispheres of the SE+SAH group relative to the SE+Sham group. A similar trend was observed between AE+SAH and AE+Sham groups; however, the AE+SAH group showed significantly reduced cerebral edema compared to the SE+SAH group (Fig. 1D). Histological evaluation via HE staining revealed substantial tissue damage in the hippocampus and cortex of SE+SAH animals, which was markedly attenuated in the AE+SAH group (Fig. 1O). Nissl staining further indicated disorganized or lost neuronal architecture and faint or dissolved Nissl bodies in the SE+SAH group, whereas AE+SAH mice exhibited preserved neuronal integrity, suggesting a protective effect of exercise (Fig. 1P).

To further assess neuronal survival, neuronal nuclei (NeuN)/terminal deoxynucleotidyl transferase-mediated biotinylated dUTP nick end labeling (TUNEL) immunofluorescence staining confirmed extensive cortical neuronal death in SAH mice, which was significantly mitigated by AE (Fig. 1E and F). At the molecular level, Western blot analysis showed that SAH induction markedly up-regulated pro-inflammatory mediators (Il-1b, Il-6, and Nos2), while AE promoted the expression of anti-inflammatory factors (Il-4, Il-10, and Tgfb1) post-SAH (Fig. 1H to N and Data S1).

### AE modulates microglial differentiation via TGF-β signaling

**Fig. 2.**
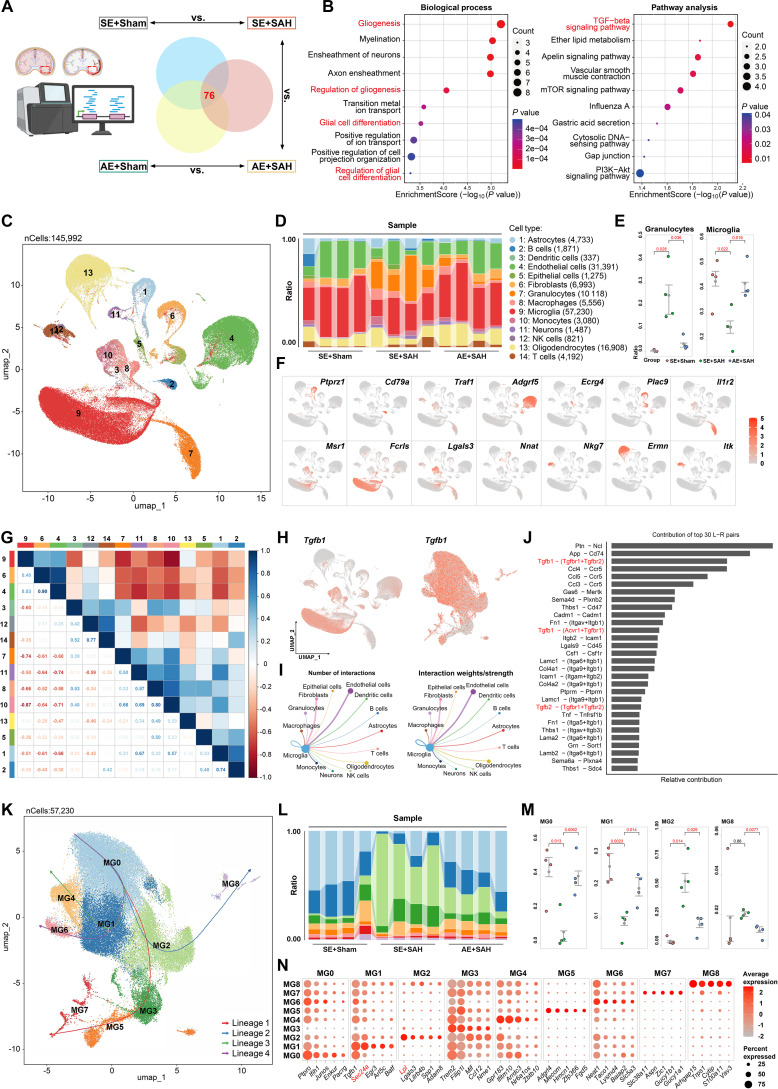
ScRNA-seq showing clusters localization and differentiation pathways of microglia subtypes in SAH. (A) RNA-seq grouping and comparison schematic diagram. Some elements are created in BioRender (2026); https://BioRender.com/ix4vz79. (B) The GO and KEGG enrichment analysis results for DEGs of RNA-seq. (C) The UMAP map of scRNA-seq of brain tissue in experimental animals. (D) Cluster percentage distribution of each sample group. (E) Differences in the distribution of granulocyte and microglia proportions between groups. (F) Marker genes of different clusters. (G) Correlation analysis and visualization of cluster proportion. (H) UMAP graphs of Tgfb1 expression. (I) Schematic diagram of interactions between multi-clusters and microglia in CellChat. (J) Importance ranking of microglial interaction pathways (ligands and receptors pairs). (K) The UMAP map of microglia. (L) Ratio distribution of microglial subtypes of each sample group. (M) Differences in the distribution of microglial subtypes proportions between groups. (N) Highly variable markers of microglial subtypes.

To elucidate the molecular mechanisms underlying the neuroprotective effects of AE against SAH-induced injury, we performed whole-transcriptome RNA sequencing (RNA-seq) across experimental groups. Comparative analysis identified 3,547 differentially expressed genes (DEGs) between SE+Sham and SE+SAH groups, 2,128 DEGs between AE+Sham and AE+SAH groups, and 1,347 DEGs between SE+SAH and AE+SAH groups, with 76 genes coexpressed across comparisons (Fig. 2A and Data S2). Gene Ontology (GO) annotation revealed that biological processes such as “gliogenesis”, “regulation of gliogenesis”, “glial cell differentiation”, and “regulation of glial cell differentiation” were prominently enriched among these DEGs (Fig. 2B). Kyoto Encyclopedia of Genes and Genomes (KEGG) pathway analysis further highlighted a central role for the TGF-β signaling pathway in mediating the protective effects of AE against SAH-induced neuronal injury (Fig. 2B).

To delineate the intrinsic mechanism by which AE modulates microglial activation, we conducted scRNA-seq on cortical tissues from the peri-hemorrhagic region in wild-type (wt) C57 mice. The results of dimensionality reduction clustering and their proportion are shown in Fig. 2C and D, detailed clustering ratio data are shown in Data S3, and highly variable genes (HVGs) of clusters are provided in Data S4. We found that there were significant differences in the proportions of granulocytes and microglia among different groups (Fig. 2E and Fig. S1A), and there was also a significant negative correlation between 2 clusters (Fig. 2G), which indicates that when SAH occurs, a large number of granulocytes infiltrate the central system, leading to a significant reduction in microglia, and AE can slow down this process. Moreover, Tgfb1 is significantly expressed in microglia (Fig. 2H). CellChat analysis also found that the TGF-β pathway plays an important role in the interaction between microglia and other cells, with Tgfb1-Tgfbr1/Tgfbr2 being prominent (Fig. 2I and J).

**Fig. 3.**
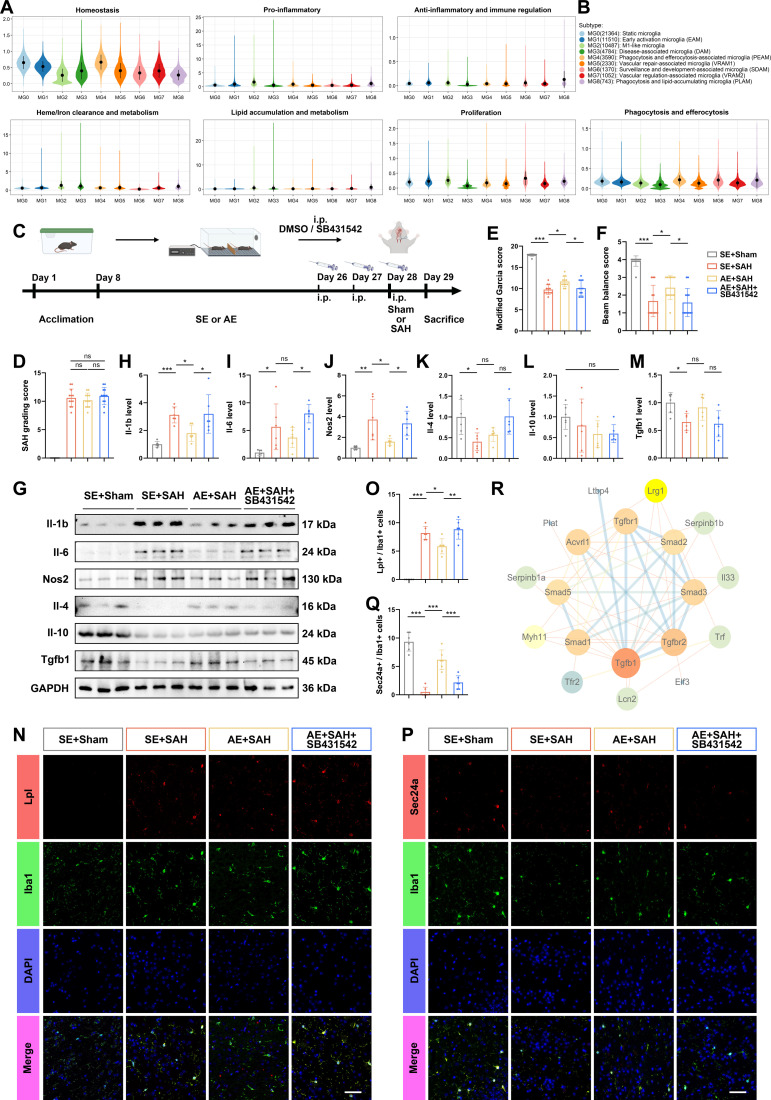
The TGF-β signaling pathway mediated the regulation of AE on microglial differentiation of SAH. (A) Multifunctional scoring of microglial subtypes. (B) Legend of microglial subtypes. (C) The complete timeline and schematic diagram of Experiment 2. (D) SAH grading score of each group in Experiment 2, *n* = 12. (E) Bars showed MGs of each group in Experiment 2, *n* = 12. (F) Bar chart of BBs in Experiment 2, *n* = 12. (G to M) Western blotting images and relative expression data demonstrating the effects of SB431542 on SAH-induced neuroinflammation following AE. *n* = 6. (N) Coronal sections of ipsilateral cortex of SAH mice followed AE with the treatment of SB431541 immunostained for Lpl (red), Iba1 (green), and DAPI (blue). Scale bars, 50 μm. (O) Quantification of the number of Lpl^+^ Iba1^+^ cells in mice at SAH 24 h. *n* = 6. (P) Coronal sections of ipsilateral cortex of SAH mice followed AE with the treatment of SB431541 immunostained for Sec24a (red), Iba1 (green), and DAPI (blue). Scale bars, 50 μm. (Q) Quantification of the number of Sec24a^+^ Iba1^+^ cells in mice at SAH 24 h. *n* = 6. Data were represented as mean ± SD. (R) Protein interaction network diagram between RNA-seq and the TGF-β signaling pathway. In (D) to (F), data were represented as median (interquartile range), and *P* values were calculated using Kruskal–Wallis *H* with Dunn test. *P < 0.05, ***P < 0.001. In (H) to (M), (O), and (Q), *P* values were calculated using one-way ANOVA with Tukey multiple comparisons. *P < 0.05, **P < 0.01, ***P < 0.001. The original Western blot images are shown in Data S1. Detailed experimental data and statistical results, including *Z* and *P* values for Kruskal–Wallis test and *F* and *P* values for ANOVAs, are provided in Data S11 to S13.

Microglia were extracted for subtype analysis, whose dimensionality reduction and differentiation trajectory are shown in Fig. 2K. The subtype proportion showed that SAH led to a significant decrease in MG0 and MG1, and a significant increase in MG2, while AE partially reversed this change (Fig. 2L and M, Fig. S1B, and Data S3; HVGs of MG subtypes are provided in Data S5). Every subtype was defined and named through characteristic genes and functional enrichment (Fig. 3A and B and Fig. S1C and D); MG0 highly expressed the classical homeostasis genes purinergic receptor P2Y12 (P2ry12) and C-X3-C motif chemokine receptor 1 (Cx3cr1), and ionized calcium-binding adaptor molecule 1 (Iba1); MG1 named early activation microglia (EAM) with high expression of Tgfb1, Sec24 homolog A (Sec24a), immediate early genes, early growth response (Egrs), and nuclear receptor subfamily 4 group A members (Nr4as); MG2 highly expressed inflammation-related genes, including lipoprotein lipase (Lpl), Il-1b, leukocyte immunoglobulin like receptor B4 beta (Lilrb4b), Spp1, and tumor necrosis factor-alpha (Tnfa); MG3 highly expressed Trem2 gene identified as disease-associated microglia; both MG4 and MG8 have significant phagocytic capabilities. MG4 focuses on efferocytosis, while MG8 emphasizes lipid regulation and anti-inflammatory effects. MG6 has a marked proliferative capacity and monitors the process of neural development. MG5 and MG7 are related to blood vessels. MG5 focuses on the repair of the BBB, while MG7 emphasizes the contraction of smooth muscles.

Thus, the above data indicate that pro-inflammatory MG2 significantly increases at SAH 24 h, while MG0 and MG1 significantly decrease. AE significantly inhibits the differentiation from MG0 to MG2. Therefore, we hypothesized that AE modulates microglial activation via the TGF-β pathway [18]. SB431542, a selective Alk5 inhibitor that blocks Tgfb1-induced Smad2/3 phosphorylation, at a dose of 10 mg/kg (i.p.), was used to test as established previously [19]. In Experiment 2 (Fig. 3C; total *n* = 65, 47 SAH-induced, 11 fatalities [23.40%], Table S1), SAH grading showed no significant difference among groups (Fig. 3D). However, SB431542 administration completely abolished the neuroprotective benefits of AE, as evidenced by worsened MGs and BBs (Fig. 3E and F).

Western blot analysis of cortical inflammatory mediators showed that AE significantly attenuated SAH-induced up-regulation of Il-1b, Il-6, and Nos2, whereas SB431542 restored their elevated expression (Fig. 3G to J). Consistent with the notion that SAH suppresses anti-inflammatory responses in the early phase, the expression levels of Il-4 and Tgfb1 were down-regulated following SAH, though not for Il-10, and AE preconditioning partially up-regulated these 2 cytokines, albeit without reaching statistical significance (Fig. 3G and K to M). Furthermore, SB431542 treatment had no significant effect on the expression of anti-inflammatory cytokines in the AE+SAH group.

Conversely, AE enhanced levels of anti-inflammatory cytokines (Il-4, Il-10, and Tgfb1), an effect suppressed by SB431542 for Il-4 and Tgfb1. Immunofluorescence quantification further demonstrated that AE reduced Lpl^+^Iba1^+^ MG2 while increasing Sec24a^+^Iba1^+^ MG1 in SAH mice—a phenotypic shift reversed by TGF-β pathway inhibition (Fig. 3N to Q). Together, these findings indicate that AE drives a microglial transition from MG2 to MG1 through activation of the TGF-β signaling pathway.

To elucidate the mechanism by which AE modulates the TGF-β pathway in microglia post-SAH, we constructed a protein–protein interaction (PPI) network using the Search Tool for the Retrieval of InteractingGenes/Proteins (STRING) database with 63 logically screened DEGs from RNA-seq data. These included 34 genes up-regulated in SE+SAH (vs. SE+Sham) but down-regulated in AE+SAH (vs. SE+SAH), and 29 genes showing the opposite trend. The resulting network visually delineated functional associations among core TGF-β pathway targets (Fig. 3R). Lrg1 emerged as the most connected node, indicated by its prominent size and color in the network, suggesting its central role in AE-mediated anti-inflammatory signaling [20].

### AE suppresses Lrg1 expression following SAH, reducing pro-inflammatory microglial differentiation and neuroinflammation

Lrg1, a secreted glycoprotein produced by hepatocytes, neutrophils, and endothelial cells, contains characteristic leucine-rich repeats and functions as a key modulator of TGF-β signaling, though it can also engage other receptors such as latrophilin-2 [21,22]. It is implicated in angiogenesis, inflammation, fibrosis, and cellular processes, including apoptosis resistance, immune evasion, and endothelial dysfunction [23–25]. Its elevated expression across pathological contexts—such as cancer, diabetic complications, and cardiovascular diseases—highlights its potential as a biomarker [26,27] and therapeutic target [28].

**Fig. 4.**
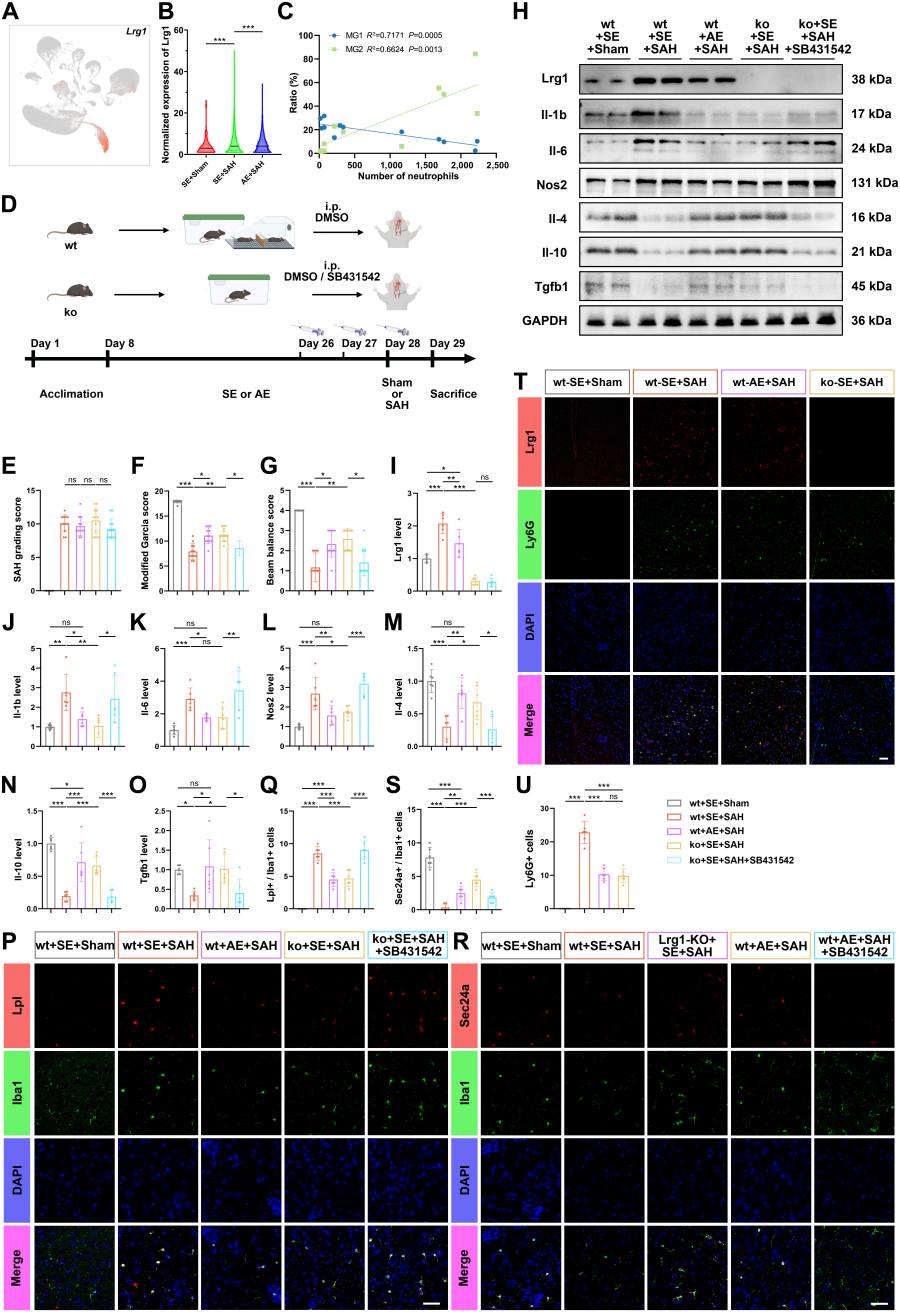
AE modulates microglial differentiation and neuroinflammation via the Lrg1/TGF-β signaling pathway. (A) Subcellular localization of lrg1 protein in scRNA-seq UMAP maps. (B) Violin diagram of Lrg1 expression in groups. *F* = 17.41, *P* < 0.001. (C) Correlation analysis and visualization of neutrophil numbers with proportional distribution of microglial subtypes. (D) The complete timeline and schematic diagram of Experiment 3. (E) SAH grading score of each group in Experiment 3, *n* = 12. (F) Bars showed MGs of each group in Experiment 3, *n* = 12. (G) Bar chart of BBs in Experiment 3, *n* = 12. (H to O) Western blotting images and relative expression data suggested that AE regulated Lrg1 expression influencing the microglial inflammatory cytokine expression. *n* = 6. (P) Coronal sections of ipsilateral cortex of the SAH model from wild-type C57 and knockout mice immunostained for Lpl (red), Iba1 (green), and DAPI (blue). Scale bars, 50 μm. (Q) Quantification of the number of Lpl+ Iba1+ cells in mice at SAH 24 h. *n* = 6. (R) Coronal sections of ipsilateral cortex of SAH model from wild-type C57 and knockout mice immunostained for Sec24a (red) and Iba1 (green), and DAPI (blue). Scale bars, 50 μm. (S) Quantification of the number of Sec24a+ Iba1+ cells in mice at SAH 24 h. *n* = 6. (T) Immunofluorescence staining of Lrg1 (red), Ly6G (green), and DAPI (blue) in the ipsilateral cortical coronal section of wild-type and knockout mice following SAH modeling. *n* = 6. Scale bars, 50 μm. (U) Quantification of the number of Lrg1+ Ly6G+ cells of wild-type and knockout mice at SAH 24 h. *n* = 6. In (E) to (G), data were represented as median (interquartile range), and *P* values were calculated using Kruskal–Wallis *H* with Dunn test. *P < 0.05, ***P < 0.001. In (I) to (O), (Q), (S), and (U), data were represented as mean ± SD. *P* values were calculated using one-way ANOVA with Tukey multiple comparisons. *P < 0.05, **P < 0.01, ***P < 0.001. The original Western blot images are shown in Data S1. Detailed experimental data and statistical results, including *Z* and *P* values for Kruskal–Wallis test and *F* and *P* values for ANOVAs, are provided in Data S14 to S16.

ScRNA-seq analysis identified neutrophils as the primary source of Lrg1 expression in the brain (Fig. 4A). The normalized expression of Lrg1 in neutrophils showed significant differences between the SE+SAH group and the AE+SAH group (Fig. 4B); both RNA-seq and WB confirmed that the SE+SAH group expressed more Lrg1 genes or proteins (Figs. 2A and 4H and I). We next investigated whether AE modulates microglial differentiation and neuroinflammation via the Lrg1/TGF-β pathway. In Experiment 3 (Fig. 4D; 45 wt and 34 Lrg1-knock out [ko] mice, 61 SAH-induced, 13 fatalities [21.31%]; Table S1), there was no difference in SAH grading score among the groups (Fig. 4E). AE significantly suppressed SAH-induced Lrg1 protein up-regulation (Fig. 4H and I). Genetic deletion of Lrg1 improved neurobehavioral outcomes (MGs and BBs) compared to wt SAH controls, whereas SB431542 administration abolished this benefit in Lrg1-ko animals (Fig. 4F and G).

Western blot analysis demonstrated that Lrg1-ko reduced pro-inflammatory markers (Il-1b, Il-6, and Nos2) and enhanced anti-inflammatory factors (Il-4, Il-10, and Tgfb1; Data S1). These effects were reversed by SB431542 (Fig. 4H and J to O). Consistently, immunofluorescence quantification showed that Lrg1-ko mice exhibited fewer Lpl^+^ Iba1^+^ MG2 microglia and more Sec24a^+^ Iba1^+^ MG1, a shift negated by TGF-β pathway inhibition (Fig. 4P to S). Collectively, these data indicate that AE restrains neuroinflammation and microglial pro-inflammatory differentiation via Lrg1-dependent regulation of TGF-β signaling.

### ScRNA-seq data suggested that mature neutrophils highly expressed Lrg1, involved in neutrophil adhesion

Based on the results from the previous section, we concluded that the number of neutrophils in the SAH had a significant negative correlation with the number of MG1 and a significant positive correlation with MG2 in correlation analysis (Fig. 4C). To further clarify the role of Lrg1 in the pathogenic mechanism of neutrophils in the SAH model, we extracted and analyzed neutrophils from scRNA-seq data (Fig. S2A). A total of 9 subtypes were identified and named according to their function (Fig. S2G). Neu1, Neu2, and Neu3 accounted for a high proportion (Data S3; HVGs of Neu subtypes are provided in Data S6). The SE+Sham group had minimal cell counts with mature Neu1 constituting the largest proportion, the SE+SAH group was dominated by mature Neu1 and hypoxia-induced Neu2, and the AE+SAH group was dominated by innate immune Neu3 (Fig. S2C). Analysis of the proportions of Neu2 and Neu3 revealed statistically significant differences between the SE+SAH and AE+SAH groups (Fig. S2B). Correlation analysis revealed that Lrg1 expression was significantly positively correlated with Neu1 and significantly negatively correlated with Neu0 and Neu3 (Fig. S2D). Simultaneously, we used the Monocle 3 software package to calculate trajectory paths of biological progression, and performed differentiation stage ordering and pseudotime analysis on these clusters (Fig. S2E). We found that Neu0 and Neu1 were in the early and mature stages of development, respectively, while Lrg1 showed the highest expression in the mature stage, which gradually decreased as neutrophils were activated and differentiated (Fig. S2E). GO functional enrichment analysis of Neu1 hypervariable genes revealed that Lrg1 was directly involved in inter-leukocyte adhesion and adhesion to endothelial cells (Fig. S2G). Tissue dual-fluorescence staining showed that AE or Lrg1-ko markedly attenuated neutrophil infiltration in the peri-hemorrhagic cortex compared with the SE+SAH group (Fig. 4T and U).

### Lrg1 secreted by primary neutrophils differentially activates Tgfbrs of primary microglia

**Fig. 5.**
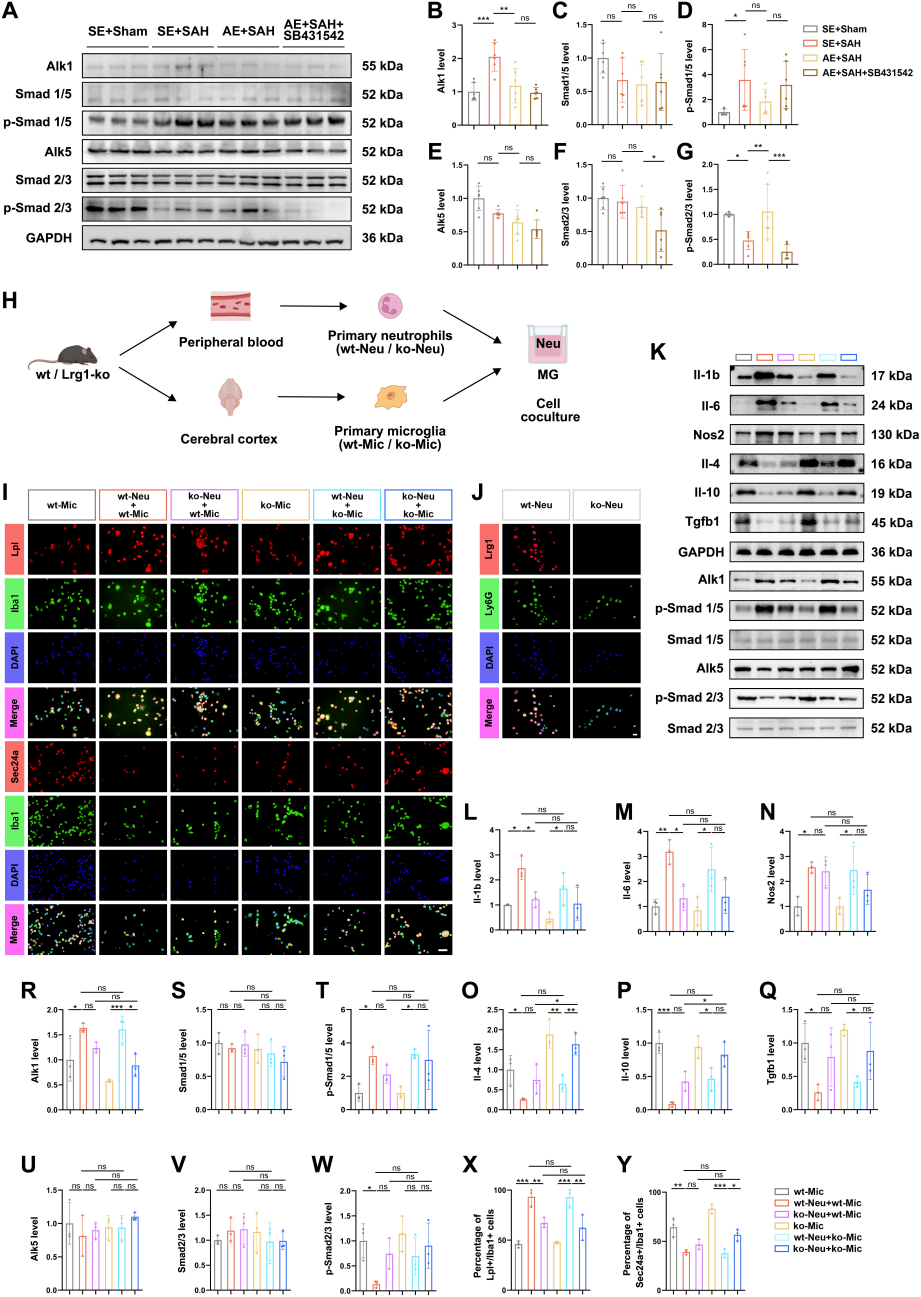
Primary peripheral neutrophil and microglia interactions based on wild-type and knockout mice. (A) Western blotting images demonstrated the effect of SB431541 treatment on the activation status of the TGF-β signaling pathway in SAH regulated by AE. (B to G) Quantitative analysis of relative expression level changes in Alk1, Alk5, Smad1/5, Smad2/3, p-Smad1/5, and p-Smad2/3 in the AE-regulated ipsilateral cortex in SAH, with the treat of SB431541. *n* = 6. (H) Schematic diagram of coculture experiments between primary peripheral neutrophils and primary microglia. (I) Immunofluorescent staining of primary microglia from wt/ko mice cocultured with primary neutrophils from wt/ko mice for Lpl/Sec24a (red), Iba1 (green), and DAPI (blue). Scale bars, 10 μm. (J) Immunofluorescent staining of primary peripheral neutrophils from wild-type and knockout mice for Lrg1 (red), Ly6G (green), and DAPI (blue). Scale bars, 10 μm. (K) Western blotting images of primary microglia from wt/ko mice cocultured with primary neutrophils from wt/ko mice on pro-inflammatory and anti-inflammatory factors and TGF-β signaling pathway. (L to W) Quantitative analysis of relative expression level of IL-1β, IL-6, Nos2, IL-4, IL-10, Tgfb1, Alk1, Alk5, Smad1/5, Smad2/3, p-Smad1/5, and p-Smad2/3 in the primary microglia cocultured with peripheral neutrophils. *n* = 3. (X) Quantitative analysis of the proportion of Lpl+/Iba1+ cells in the immunofluorescent staining of primary microglia in the primary cell coculture experiment. *n* = 3. (Y) Quantitative analysis of the proportion of Sec24a+/Iba1+ cells in the immunofluorescent staining of primary microglia in the primary cell coculture experiment. *n* = 3. In (B) to (G) and (L) to (Y), data were represented as mean ± SD, and *P* values were calculated using one-way ANOVA with Tukey multiple comparisons. *P < 0.05, **P < 0.01, ***P < 0.001. The original Western blot images are shown in Data S1. Detailed experimental data and statistical results, including *F* and *P* values for ANOVAs, are provided in Data S17 to S19.

Western blot analysis of mice hemorrhagic cortex samples further elucidated TGF-β pathway dynamics. The SE+SAH group displayed elevated activin a receptor like type 1 (Alk1) levels and increased Smad1/5 phosphorylation, accompanied by reduced Smad2/3 phosphorylation—a pattern attenuated by AE (Fig. 5A, B, D, and G and Data S1). In contrast, neither SAH induction nor AE intervention significantly altered the total protein levels of Alk5, Smad1/5, or Smad2/3 (Fig. 5A, C, E, and F and Data S1). Notably, administration of the Alk5 inhibitor SB431542 partially reversed the AE-mediated suppression of Smad1/5 phosphorylation and robustly inhibited Smad2/3 activation (Fig. 5A, D, and G), confirming the functional involvement of TGF-β receptor signaling in AE-induced neuroprotection.

To define the specific role of Lrg1 in neutrophil–microglia crosstalk, we established a coculture system combining primary neutrophils and microglia from either wt or Lrg1-ko mice (Fig. 5H). This design enabled independent assessment of how Lrg1 deletion in either cell type influences microglial differentiation and inflammatory signaling.

Notably, wt-neutrophils consistently induced microglial pro-inflammatory differentiation—regardless of the microglial genotype—elevating pro-inflammatory mediators (Il-1b, Il-6, and Nos2) and enhancing Alk1–Smad1/5 signaling, while suppressing anti-inflammatory factors (Il-4, Il-10, and Tgfb1) and Alk5–Smad2/3 activation (Fig. 5K to W and Data S1). In contrast, Lrg1-ko neutrophils significantly attenuated these pro-inflammatory responses in wt-microglia and partially restored anti-inflammatory signaling and Smad2/3 phosphorylation, though some changes did not reach statistical significance.

Phenotypically, wt-neutrophils increased the proportion of Lpl^+^ Iba1^+^ microglia, whereas Lrg1-ko neutrophils markedly reduced pro-inflammatory differentiation and promoted Sec24a^+^Iba1^+^ microglial expansion (Fig. 5I, X, and Y). Together, these results demonstrate that neutrophil-derived Lrg1 directly promotes microglial pro-inflammatory differentiation and sustains neuroinflammation via biased activation of the Alk1–Smad1/5 pathway.

### Lrg1 transduced Alk5/Alk1 receptor signals via Tgfb1 and Eng

**Fig. 6.**
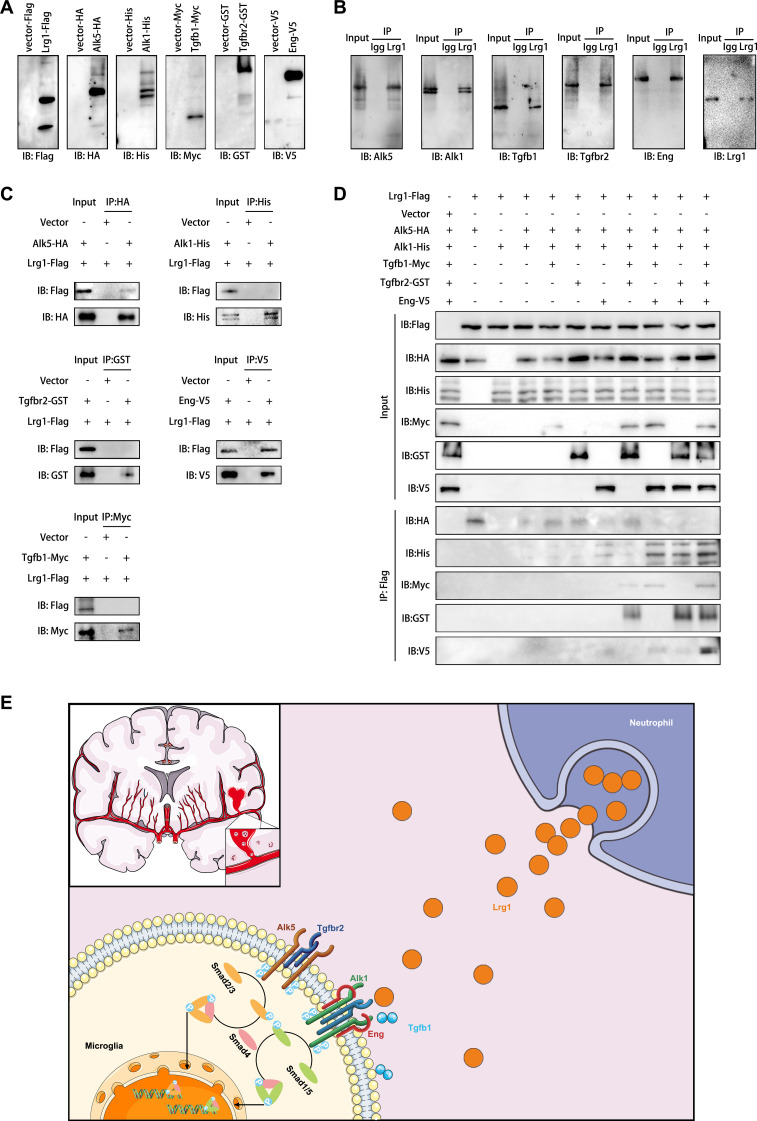
Lrg1 interacts with the TGF-β signaling pathway. (A) Immunoprecipitation of media containing individual proteins or extracellular domains of TGF-b pathway receptors, produced in separately transfected HEK293T cells cultured by serum-free media. (B) Immunoprecipitation (IP) of Lrg1 from primary wild-type microglia lysates, cocultured with primary neutrophils and coprecipitates Alk1, Alk5, Tgfb1, Tgfbr2, and ENG. Homologous IgG antibody in cocultured primary wild-type microglia lysates did not coprecipitate Alk1, Alk5, Tgfb1, Tgfbr2, and ENG. (C) Immunoprecipitation of the corresponding peptide-tagged antibody added to HEK293T cells cotransfected with lrg1 (Flag-tag) and every receptor extracellular domain plasmid respectively, indicating which direct interactions with Alk5 (HA-tag) and Eng (V5-tag). Immunoprecipitation of Alk1 (His-tagged), Tgfb1 (Myc-tag), or Tgfbr2 (GST-tag) in the presence of Lrg1 did not coprecipitate the former. (D) Medium containing full-length Lrg1 protein (Flag-tagged) was incubated in vitro with specific combinations of media containing TGF-β receptors extracellular domains or Tgfb1 for immunoprecipitation of Flag-tag and validation. (E) Graphical abstract of communicating mechanisms between neutrophil and microglia through the Lrg1-TGF-β signaling pathway in SAH. SAH, subarachnoid hemorrhage; Lrg1, leucine-rich alpha-2-glycoprotein 1; Tgfb1, transforming growth factor-beta 1; Alk1 (Acvrl1), activin A receptor like type 1; Alk5 (Tgfbr1), transforming growth factor beta receptor 1; Tgfbr2, transforming growth factor beta receptor 2; Eng, endoglin; Smad1/5, Smad family member 1/5; Smad2/3: Smad family member 2/3.

Based on previous reports implicating endothelial Lrg1 in aberrant vascular proliferation, we hypothesized that neutrophil-derived Lrg1 may directly interact with microglial Alk1 or Alk5 receptors. Co-immunoprecipitation (Co-IP) assays following coculture of wt microglia and neutrophils confirmed that Lrg1 immunoprecipitates contained Alk1, Alk5, Tgfb1, Tgfbr2, and endoglin (Eng) (Fig. 6B), suggesting that Lrg1 may modulate TGF-β signaling by fine-tuning the stoichiometry of TGF-β receptor complexes.

To validate the specific binding of the ligand–receptor complex, we transfected human embryonic kidney 293T (HEK293T) cells with plasmids expressing full-length Lrg1 and the extracellular domain of the TGF-β receptor (Fig. 6A). Immunoprecipitation confirmed that Lrg1 directly and specifically binds to Alk5 and Eng, but not to Alk1 alone (Fig. 6C). Considering that Lrg1 could activate Alk1 and downstream Smad1/5 phosphorylation, we hypothesized that Lrg1–Alk1 interaction requires an additional mediator. Wang et al. [29]. demonstrated that Eng was a prerequisite for Lrg1–Alk1 binding, and Tgfb1 or Tgfbr2 could accelerate this process in endothelial cells. Therefore, using serum-free medium, we constructed a mixed combination of Lrg1 and multiple receptors from single-plasmid transfected HEK293t cells expressing full-length Lrg1 or recombinant extracellular domains of TGF-β pathway components (Fig. 6D). In the absence of Eng, Lrg1 alone bound to Alk5 but failed to initiate intracellular signaling, although this did not fully account for the observed reduction in Alk5-Smad2/3 phosphorylation. Consistent with the work of Wang et al., we demonstrated that Eng was essential for Lrg1–Alk1 association, while Tgfb1 and Tgfbr2 enhanced this interaction and simultaneously suppressing Lrg1–Alk5 binding (Fig. 6D). These results indicated that Tgfb1 promotes the formation of the Lrg1/Alk1/Tgfbr2/Eng ligand–receptor complex, redirecting signaling from the Alk5–Smad2/3 pathway to Smad1/5 activation.

In cellular contexts where both Alk1 and Alk5 are present, Lrg1 formed heterotrimeric complexes with both receptors. Tgfb1 competed with Lrg1 for Alk5 binding, while the simultaneous presence of Tgfb1 and Tgfbr2 enabled weak Lrg1–Alk1 association, likely through a higher-order Lrg1–Tgfb1–Alk5–Alk1–Tgfbr2 polymeric assembly (Fig. 6D). Although the signaling capacity of this complex requires further validation, our findings demonstrate that Tgfb1 preferentially promotes formation of Lrg1/Alk1/Tgfbr2/Eng signaling complexes, thereby diverting TGF-β signaling from Alk5–Smad2/3 toward Alk1–Smad1/5 activation. A schematic model summarizing this mechanism is presented in Fig. 6E.

## Discussion

The present study identifies a previously unrecognized mechanism by which daily AE preconditioning confers neuroprotection against EBI following SAH: disruption of the pathogenic crosstalk between infiltrating neutrophils and resident microglia through receptor-level reprogramming of Lrg1 signaling. Our findings advance the field in several important respects. First, we uncover a novel Tgfb1^high^ early-response microglial subset whose emergence is reversely coupled to neutrophil infiltration, revealing a dynamic immune cell interaction not previously described in SAH pathophysiology. Second, we establish mature neutrophil-derived Lrg1 as a critical immune checkpoint that drives microglial activation via context-dependent engagement of the TGF-β receptor system. Third and most importantly, we demonstrate that exercise preconditioning fundamentally redirects Lrg1 binding from Alk1 to Alk5, converting a pro-inflammatory signal into a protective one, a paradigm of exercise-induced receptor reprogramming with broad therapeutic implications.

While neuroinflammation has long been recognized as a central driver of EBI after SAH, the precise cellular choreography underlying this process has remained incompletely understood. Our scRNA-seq analysis provides unprecedented resolution of this dynamic interplay. The identification of a Tgfb1^high^ early-response microglial subset, strongly inhibited with neutrophil infiltration, suggests that this population may serve as a sentinel population that maintain neuroimmune homeostasis. Tgfb1 is a pleiotropic cytokine with context-dependent pro- or anti-inflammatory effects, whereas the expression of the Tgfb1 in stroke diseases remains controversial. In the latest review, 76% studies focusing on ischemic stroke showed a protective effect of Tgfb1, and within hemorrhagic stroke studies, 33% showed a protective effect of Tgfb1, with others mainly focusing on sustained secretion from activated platelets, exacerbating fibrosis and hydrocephalus [30]. Our data support the neuroprotective effect of Tgfb1 in the acute phase of SAH; however, scRNA data showed no significant differences. Hence, we believe that the key lies not in the transcriptional level, but in post-synthetic modifications and bioactive functions of ligands. In the acute phase (post-SAH 6 to 72 h), the signaling efficacy of Tgfb1 diminished due to increased inhibitory Smads and reactive oxygen species (ROS) impairing ligand and receptor binding and signaling, and promoting receptor Alk5/Tgfbr2 degradation [30]. It is worth noting that the expression of Alk1 is completely opposite with Alk5, both type I serine/threonine kinase receptors mediating TGF-β signaling, but activates distinct downstream pathways that differentially regulates microglial differentiation states. Alk1 primarily activates Smad1/5/8 signaling, which promotes expression of pro-inflammatory genes Il-1b, Nos2 and Tnfa, through competitive inhibition of Smad2/3 signaling. High Alk1/Alk5 ratio favors pro-inflammatory differentiation with the assistance of Tgfb1 in SAH. Moreover, Alk1 strongly activates mitogen-activated protein kinase pathways that maintain microglia in the pro-inflammatory state, and Alk1 cooperates with bone morphogenetic protein receptors to enhance cerebral edema [31]. Alk5 phosphorylates Smad2/3, which forms complexes with Smad4 and translocates to the nucleus to induce M2-associated gene expression (Il-10 and Tgfb1), enhancing anti-inflammatory responses. Understanding Alk1/Alk5 balance activation is crucial for developing targeted immunomodulatory therapies in neurological disorders.

In the immediate aftermath of SAH, we propose that neutrophil-derived signals, potentially including Lrg1 itself, suppress a subset of microglia toward this Tgfb1-expressing phenotype, which in turn perpetuates the inflammatory cycle. Lrg1 was originally described as a hepatocyte-derived factor involved in erythroid differentiation and later implicated in angiogenesis through its modulation of TGF-β signaling. In recent years, some studies have regarded Lrg1 as a blood marker for the poor prognosis of inflammatory diseases, related to the increased infiltration of inflammatory cells. Wang et al. [32] found that Lrg1 promotes atherosclerosis by inducing macrophage M1-like polarization. Urushima et al. [33] discovered that the reason why Lrg1 led to differential differentiation of immune cells likely arises from Lrg1’s capacity to enhance the Il6–signal transducer and activator of transcription 3 (Stat3) signaling pathway. Notably, scRNA data indicate that Lrg1 mainly originates from peripheral neutrophils and macrophages. Coincidentally, Jiang et al. [34] found that Lrg1-enriched extracellular vesicle regulates macrophage polarization via an Alk5-dependent process promoting keratinocyte inflammation. Given that peripheral neutrophils and macrophages cannot cross the healthy BBB, the role of this interaction process in hemorrhagic stroke is easily overlooked. Ruan et al. [35] studied scRNA data of cerebral ischemia models and discovered that microglia and macrophages within ischemic regions in Lrg1-ko mice exhibited a shift from a pro-inflammatory phenotype to an anti-inflammatory, tissue repair-promoting state. Additionally, the impact of Lrg1 on microglial function extends beyond TGF-β pathway modulator. Lrg1 enhances nicotinamide adenine dinucleotide phosphate oxidase activity [36], increasing ROS generation that contributes to oxidative stress and BBB disruption [34]. Multiple transcription factors, Stat3, nuclear factor kappa-B, and peroxisome proliferators-activated receptors, stimulated in the process of microglial pro-inflammatory differentiation, selectively recognize the gene of Lrg1 promoter increasing transcription, and there is highly likely existing Lrg1-mediated pro-inflammatory positive feedback [22]. Thus, lrg1 is highly likely to be a key cytokine regulating the interaction between neutrophils and microglia in SAH, and the coculture experiments of peripheral neutrophils and microglia in this study confirmed this hypothesis. The expression of lrg1 is closely associated with neutrophil infiltration. In alkali-burned corneas, Lrg1 increased fibrogenic protein expression and neutrophil infiltration, while Lrg1-specific siRNA reduced these effects [37]. Endothelial Lrg1 directly promotes neutrophil chemotaxis and enhances C-X-C motif chemokine ligand 1 (CXCL1) secretion, a key chemokine for neutrophil recruitment [38]. Lrg1-ko studies in cerebral ischemia–reperfusion injury showed reduced neutrophil infiltration and BBB disruption and neuron death, and exhibited up-regulated tight junction proteins called Claudins [35]. Therefore, the inflammatory mediators above disrupt BBB integrity by degrading tight junction proteins and enhancing vascular permeability, facilitating the infiltration of neutrophils and macrophages, the first leukocytes recruited to the injury site, releasing additional DAMPs, matrix metalloproteinases, and ROS that collectively aggravate EBI [39].

Previous work by Wang et al. [29] systematically investigated Lrg1–Tgfbrs interactions in endothelial cells, demonstrating that Lrg1 exhibits distinct coreceptor requirements for binding: it specifically associates with Alk1 only in the presence of both Tgfb1 and Eng, while binding exclusively to Alk5 when Eng is absent, indicating competitive binding between Eng and Alk5 for Lrg1. Neither Tgfb1 nor Tgfbr2 alone influenced these interactions in the absence of Eng. However, when Eng was present, Tgfbr2 formed complexes with both Lrg1–Alk1 and Lrg1–Alk5, with Tgfb1 enhancing the former interaction while suppressing the latter. These findings establish that Lrg1 can assemble distinct receptor complexes containing Alk1, Alk5, Tgfbr2, and Eng, with the Lrg1/Alk1/Tgfbr2/Eng complex predominating in the presence of Tgfb1, thereby modulating the TGF-β signaling output. This receptor-level reprogramming has important implications for understanding how behavioral interventions confer disease resistance. Rather than simply dampening inflammation globally, which could impair essential immune functions, AE appears to recalibrate the signaling circuitry, represents a more sophisticated form of neuroimmune modulation than simple inhibition, and may explain why exercise preconditioning is associated with improved outcomes across diverse neurological insults without apparent immunosuppressive side effects. Consistent with this regulatory role, Lrg1 has been implicated in diverse pathological contexts including diabetic kidney disease, wound healing [40], epithelial–mesenchymal transition in gastrointestinal cancer [41], and fibrotic diseases [42], positioning it as a promising therapeutic target.

In contrast to molecular translation, the implementation of AE is poised to elicit broader translational interest. While no standardized clinical framework currently exists for exercise protocols in this context, the neuroprotective effects observed in our study, together with supportive evidence from the literature, provide initial clinically relevant parameters for individuals at risk of SAH. In ischemic stroke models, comparable moderate-intensity forced exercise regimens have been consistently shown to attenuate infarct volume and improve functional recovery, suppressing neutrophil chemotaxis and NETs, and improving acute outcomes after ischemic stroke [43,44]. Merging evidence extends this protection to hemorrhagic stroke, and the regimen that yielded pronounced neuroprotection in our study comprised 3 key elements: moderate intensity (treadmill speeds of 5 to 15 m/min, corresponding to approximately 60% to 70% of maximal oxygen uptake in mice), progressive overload (weekly increments in speed and duration), and sufficient frequency (for a minimum duration of 3 weeks) [45,46]. Notably, the observed protective effect was associated with a cumulative volume of moderate-intensity endurance exercise. When extrapolated to humans, this corresponds to approximately 150 to 200 min of moderate-intensity aerobic activity per week (e.g., brisk walking, jogging, or cycling at 60% to 70% of heart rate reserve), which aligns with current physical activity guidelines for cardiovascular health [47,48].

Nevertheless, several inherent limitations warrant careful consideration. First, the current protocol remains largely empirical, and alternative exercise modalities, such as voluntary wheel running, may produce different effects. The influence of different exercise styles, intensities, and durations on SAH-induced EBI remains to be determined. Second, standardized frameworks for evaluating the cross-species consistency of exercise paradigms remain lacking. Consequently, any recommendations for human health must await prospective clinical validation, which currently precludes the establishment of evidence-based exercise prescriptions for clinical populations [49]. Third, marked interindividual variability in exercise response warrants caution, especially in unruptured aneurysm carriers. Computational fluid dynamics suggests that exercise-induced elevations in heart rate and flow velocity may increase wall shear stress, potentially modulating rupture risk [50]. Notwithstanding these concerns, moderate-intensity aerobic activity remains safe for low-risk individuals and confers established cardiovascular benefits [47]. Collectively, these findings support a biological rationale for structured, moderate-intensity aerobic prehabilitation in high-risk populations and inform future dose–response studies in animal models.

Aside from the specifics of the AE, several limitations of this study should be acknowledged. First, our assessment was confined to the acute phase (24 h post-SAH), leaving the durability of exercise-induced receptor reprogramming and its impact on long-term outcomes including delayed ischemia, chronic neuroinflammation, and cognitive dysfunction unexplored. Also, the exclusive use of young male C57 mice limit the generalizability of our findings across sexes, ages, and genetic backgrounds. Second, the neutrophil–microglia coculture system in vitro oversimplifies the complex multicellular microenvironment of the injured brain. The contribution of other cell types, particularly astrocytes and endothelial cells, to the neutrophil–microglia axis and their modulation by exercise warrant further investigation. Finally, the biological mechanism of Lrg1 protein remains incompletely established, as serum Lrg1 primarily derived from neutrophils undergoes differential glycosylation that may modulate TGF-β pathway-mediated microglial activation or neutrophil infiltration [21]. Moreover, the precise mechanisms by which AE regulates peripheral neutrophil or systemic immune status require further elucidation.

In short, our study demonstrates that a 3-week preconditioning regimen of AE significantly mitigates acute neurological deficits, cerebral edema, neuronal apoptosis, and neuroinflammation measured at 24 h post-SAH. Mechanistically, we found that infiltrating peripheral neutrophils exacerbate EBI by releasing Lrg1, which skews microglial TGF-β signaling toward the pro-inflammatory Alk1 axis, thereby promoting pro-inflammatory differentiation while suppressing the Tgfb1^high^ expression subtype. AE intervention attenuates this cascade by reducing neutrophil recruitment and Lrg1 expression, thereby rebalancing microglial differentiation toward the anti-inflammatory Alk5 pathway and alleviating neuroinflammation. Collectively, these findings identify AE as a promising nonpharmacological strategy for prophylactic attenuation of SAH-induced EBI.

## Materials and Methods

### Study design

This study comprised 3 experimental sections, with the grouping and exclusion numbers for each section detailed in Table S1. Prior to animal experiments, mice were randomly assigned to either the AE group or the SE group and were acclimated for 7 days in specific pathogen-free grade individually ventilated cages at the Animal Experiment Center of Renmin Hospital of Wuhan University, with environment maintained controlled humidity (65.0% ± 5.0%) and temperature (25.0 ± 1.0 °C) under a 12-h light/dark cycle, and unrestricted access to food and water for at least 1 week. Subsequently, the mice in the AE group underwent 3 weeks of forced treadmill exercise, and SE groups were allowed to move freely in their cages; the SAH model was established following exercise cessation, as described below.

### Experimental animals

Wt C57BL/6J male mice (8 to 10 weeks weighing 18 to 22 g) were obtained from the Central Laboratory of Renmin Hospital of Wuhan University. Conventional Lrg1 knockout mice on a C57BL/6 background were originally generated and mated at Cyagen Biosciences Corporation (strain name: C57BL/6NCya-*Lrg1^em1^*/Cya, Serial Number: KOCMP-76905-Lrg1-B6N–VA). Two exons were identified, with the “ATG” start codon in exon 1 and the “TGA” stop codon in exon 2 (Transcript Lrg1-201: ENSMUST00000041357). Exons 1 and 2 were selected as the target region, which contains a 1,029-base pair (bp) coding sequence. The offspring underwent genotyping via polymerase chain reaction (PCR) and was reared to 8 weeks age for breeding or experimental purposes.

### Mouse AE model

AE was performed on customized treadmills with a 5° incline and a stable transmission rate, separated into independent spaces with partitions for small animals to avoid mutual disturbance. All the mouse exercises were carried out from 6 PM to 10 PM on the same day to minimize diurnal effects. A 3-day treadmill acclimation (5 m/min, 10 min/day) was performed prior to formal training to identify and exclude noncompliant mice. The official AE program employed a widely accepted approach that ensured steady increases in speed and incorporated appropriate resistance training [51,52]. In short, the mice were trained at speeds of 5 and 10 m/min for 15 min each in the first week, at speeds of 5, 10, and 12 m/min for 10 min each in the second week, and at speeds of 5, 10, and 15 m/min for 10 min each in the third week.

### SAH model and grading

The murine SAH model was established using the endovascular perforation technique as previously described [53]. Briefly, mice were anesthetized with isoflurane gas at an initial induction concentration of 4%. Once anesthetized, each mouse was placed in the supine position on a thermostatically controlled operating table maintained at 37.0 ± 0.5 °C, and anesthesia was maintained with 2% isoflurane delivered continuously through a face mask secured over the head. The left common carotid, external carotid (ECA), and internal carotid (ICA) arteries were exposed through a midline cervical incision and properly ligated. A 4-0 monofilament suture was introduced through the ECA into the ICA until resistance indicated vascular perforation, then immediately withdrawn. The ECA was subsequently ligated and the surgical incision was closed. Sham-operated animals underwent identical procedures excluding vascular perforation. SAH severity was assessed blindly at euthanasia using an established 18-point grading system [53]. The basal subarachnoid cistern was divided into 6 segments, each scored 0 to 3 based on hemorrhage extent: 0 (none), 1 (minimal), 2 (moderate clot with visible arteries), and 3 (massive clot obscuring arteries). Mice with a total score below 8 were excluded from the study.

### Neurological scoring

Neurological function was assessed 24 h post-SAH using the MGs and BBs. The MGs evaluated 6 parameters: spontaneous activity, axial symmetry, forelimb extension, climbing, trunk proprioception, and vibrissae touch. Each parameter was scored from 0 to 3, yielding a total score of 0 to 18. The BBs quantified beam-walking ability over 60 s using a 4-point scale: 0 (falls immediately), 1 (remains stationary on beam), 2 (walks but falls), 3 (walks < 20 cm), and 4 (walks ≥ 20 cm). Higher scores in both tests indicated better neurological performance.

### Brain water content measurement

Mice were deeply anesthetized with isoflurane gas, then rapidly decapitated. Brain tissue, divided into left and right hemispheres, was collected at SAH 24 h, and was weighed immediately to obtain the wet weight succeeded by drying at 105 °C for 24 h to determine the dry weight. The percentage of brain water content was calculated using the following formula: [(wet weight − dry weight)/wet weight] × 100%.

### Histology and immunohistochemistry

At 24 h post-SAH, mice were deeply anesthetized with isoflurane and placed in the supine position on a surgical board. A midline incision was made in the chest to expose the heart, and transfusion needle was inserted into the left ventricle and secured for transcardial perfusion with 60 ml of ice-cold isotonic normal saline or phosphate-buffered saline (PBS) followed by 30 ml of 4% paraformaldehyde (PFA). Brains were harvested and immersion-fixed in 4% PFA in 0.1 M phosphate buffer (pH 7.4) overnight at room temperature, subsequently embedded in paraffin, and coronally sectioned at 4 μm thickness. For histological assessment, sections were stained with hematoxylin and eosin (H&E) to evaluate general neuropathology, while Nissl staining was employed to detect specific neuronal damage in the hippocampus and cortical regions adjacent to the hemorrhage.

### Immunofluorescence staining

Double immunofluorescence staining was performed according to the protocol described in previous studies [54]. After deparaffinization and rehydration, endogenous peroxidase was blocked with methanol containing 0.9% hydrogen peroxide for 15 min. The sections were rinsed with PBS (pH 7.6) 3 times for 10 min each and were then blocked with 10% skim milk in PBS for 20 min. The brain tissue sections were incubated individually at 4 °C overnight with the following primary antibodies: Goat anti-ionized calcium-binding adaptor molecule 1 (Iba1) monoclonal antibody (Abcom, ab289874, diluted 1:1,000); Rabbit anti-Sec24 homolog A (Sec24a) polyclonal antibody (Proteintech, 15958-1-AP, diluted 1:200); Mouse anti-lipoprotein lipase (Lpl) monoclonal antibody (Santa, sc-373759, diluted 1:100); Rabbit anti-Lrg1 polyclonal antibody (Proteintech, 13224-1-AP, diluted 1:100); and Rat anti-lymphocyte antigen 6 family member G (Ly6G) monoclonal antibody (Cell Signaling Technology, CST, #68590, FITC conjugate, diluted 1:100). Subsequently, the sections were washed 3 times in PBS for 10 min each and then incubated at room temperature for 1 h with the appropriate secondary antibody: Donkey anti-mouse IgG conjugated to Alexa Fluor 594 (Antgene, ANT029, diluted 1:400); Donkey anti-rabbit IgG conjugated to Alexa Fluor 594 (Antgene, ANT030, diluted 1:400); and Donkey anti-goat IgG conjugated to Alexa Fluor 488 (Antgene, ANT025, diluted 1:400). The sections were then visualized under a fluorescence microscope and photographed. Coronal sections revealing the same region adjacent to the hemorrhagic cortex were randomly selected. Lpl^+^Iba1^+^, Sec24a^+^Iba1^+^, and Lrg1^+^Ly6G^+^ cells were detected and counted in these regions. The data are expressed as the number of cells/fields.

### TUNEL assay

After fixation, the tissue was processed for TUNEL assay with the cell apoptosis detection kit (Servicebio, G1502-50T), and Rabbit anti-NeuN polyclonal antibody (ABclonal, A0951) and its corresponding secondary antibody: Donkey anti-rabbit IgG conjugated to Alexa Fluor 488 (Antgene, ANT024, diluted 1:400). TUNEL^+^NeuN^+^ cells were morphologically recognized as apoptotic neurons. Coronal sections from the same region of the cortex ipsilateral to the vessel puncture site were randomly selected to count. Imaging was performed using a fluorescence microscope at 20× magnification to detect the number of double-positive cells.

### RNA extraction and RNA-seq

The mice were deeply anesthetized and perfused after SAH 24 h, and the cerebral cortex was rapidly dissected on ice, rinsed with ice-cold PBS to remove residual blood, and transferred to RNase-free 1.5-ml microcentrifuge tubes. Total RNA was extracted using TRIzol reagent (Shanghai Majorbio Biopharm Technology Co., Ltd., China) according to the manufacturer’s protocol. RNA integrity was assessed on a 5300 Bioanalyzer (Agilent), and concentration/purity were measured with an ND-2000 spectrophotometer (NanoDrop Technologies). Only high-quality RNA samples (OD260/280: 1.8 to 2.2, OD260/230 ≥ 2.0, RQN ≥ 6.5, 28S:18S ≥ 1.0, yield > 1 μg) were used for library construction.

Sequencing libraries were prepared using the Illumina Stranded mRNA Prep Ligation kit (Illumina, San Diego, CA). Briefly, 1 μg of total RNA was subjected to poly(A) mRNA selection with oligo(dT) beads, followed by fragmentation. Double-stranded cDNA was synthesized with random hexamer primers (Illumina, USA) using the SuperScript double-stranded cDNA synthesis kit (Invitrogen, USA). The cDNA product underwent end-repair, phosphorylation, and “A” base addition according to Illumina’s protocol. Fragments of approximately 300 bp were size-selected on 2% Low Range Ultra Agarose gels, followed by 15 cycles of PCR amplification with Phusion DNA polymerase (NEB). Libraries were quantified on a Qubit 4.0 and sequenced on a NovaSeq X Plus platform (2 × 150 bp read length).

Raw reads were trimmed and quality-controlled using fastp (v0.19.5) with default parameters. Clean reads were aligned to the reference genome (orientation-aware mode) using HISAT2 (v2.1.0). Mapped reads were assembled by StringTie (v2.1.2) in a reference-guided manner.

Differential expression analysis was performed with DESeq2 (v1.38.0). Genes with |log2FC| ≥ 1 and FDR ≤ 0.05 were considered significantly differentially expressed. Functional enrichment analyses (GO and KEGG pathway) were conducted using Goatools (v0.6.5) and KOBAS (v2.1.1), and significantly enriched terms were defined at a Benjamini-corrected *P* value ≤ 0.05.

### ScRNA-seq

Single-cell libraries were prepared using the 10× Genomics Chromium platform according to the manufacturer’s protocol. Briefly, a sorted single-cell suspension was mixed with 10× barcoded gel beads and oil, then loaded onto a Chromium Single Cell B Chip. The Chromium Controller generated nanoliter-scale Gel Bead-in-Emulsions (GEMs), each encapsulating a single cell. Full-length cDNA was synthesized by incubating GEMs in a thermal cycler. After breaking the GEMs, pooled cDNA was cleaned with DynaBeads MyOne Silane beads and preamplified by PCR. Sequencing libraries were constructed through cDNA fragmentation, end-repair and A-tailing, SPRIselect size selection, adapter ligation, sample index PCR amplification, and a final SPRIselect clean-up. Libraries were sequenced on a NovaSeq 6000 (Illumina) with paired-end 150-bp reads and a 14-cycle index read. Raw BCL files were converted to FASTQ using Cell Ranger v7.0.0, and reads were aligned to the mouse reference genome (refdata-gex-mm10-2020-A). Cell Ranger also generated a gene-barcode expression matrix.

Downstream analysis was performed in R (v4.2). Doublets were predicted and removed using scDblFinder (v1.12.0). Cells were filtered to retain those with at least 200 detected genes, and outliers (top and bottom 2% quantiles) based on gene count or unique molecular identifier (UMI) count were excluded. Cells with mitochondrial read proportion >20% were discarded. Only cells with a cellular complexity score (Log10GenesPerUMI) >0.80 were retained. Genes expressed in fewer than 10 cells were removed.

The Seurat package (v4.3.0) was used for subsequent analysis. Raw counts were normalized using LogNormalize with a scale factor of 10,000. HVGs were identified with FindVariableFeatures (default parameters). Data were scaled and centered using ScaleData with a negative binomial model, regressing out the percentage of mitochondrial genes, cell cycle effects, and UMI counts. Principal component analysis was performed on HVGs; significant principal components were selected based on the elbow of standard deviations and the JackStraw test. Nonlinear dimensional reduction and 2-dimensional projection were achieved with UMAP, followed by graph-based clustering. Cluster-specific marker genes were identified using FindAllMarkers, and clusters were annotated based on canonical cell-type markers.

### Extraction and coculture of primary cells

Primary microglial cultures were prepared from neonatal mice (postnatal 1 to 3 days) as previously described [55]. Cerebral cortices were dissected, meninges were removed, and tissues were dissociated enzymatically with 0.005% trypsin/ethylenediaminetetraacetic acid (EDTA) and mechanically triturated. Cells were seeded into T25 flasks at a density of 2 to 5 × 10^6^ cells per flask and maintained in Dulbecco’s Modified Eagle Medium/Nutrient Mixture F-12 (DMEM/F12, Cellmax, CGM105.05) medium supplemented with 10% fetal bovine serum (FBS, Cellmax, SA211.02) and 1% penicillin/streptomycin (Procell, PB180120) at 37 °C with 5% CO₂. Medium was refreshed every 2 to 3 days. From day 7 onward, 5 ng/ml granulocyte-macrophage colony stimulating factor (MCE, HY-P70623) was added to promote microglial proliferation. Microglia were harvested by mechanical shaking at 200 revolutions per minute (rpm) for 60 min once confluent. Immunocytochemistry using goat anti-Iba1 antibodies (Abcom, ab289874) confirmed microglial identity.

Primary mouse peripheral blood neutrophils were extracted using the kit (TBDscience, LZS1100). In brief, freshly peripheral blood from 3 to 4 mice was collected into EDTA anticoagulant tube, and neutrophils were subsequently isolated by erythrocyte sedimentation and discontinuous gradient centrifugation, as previously described [56]. Primary neutrophils were cultured in Roswell Park Memorial Institute-1640 (RPMI 1640, Gibco, C11875500BT) medium supplemented with 10% FBS (Cellmax, SA211.02), and used for coculture within 24 h after extraction. Rat anti-Ly6G (FITC Conjugate) antibodies (CST, #68590) were used to identify neutrophils by immunocytochemistry.

Transwell plates (Corning) were used for primary neutrophil–microglia coculture. Purely isolated microglia were implanted into the lower chamber with DMEM/F12 medium 1 day in advance. After 24 h, when microglia had partially adhered, neutrophils were seeded into the upper chamber with RPMI 1640 medium. Coculture was performed for 24 h in a humid environment at 37 °C and 5% carbon dioxide (CO₂), followed by subsequent experiments.

### PPI network building

Version 11.0 of the STRING database was downloaded from the consortium’s website and gene identifiers from RNA-seq were mapped to Stable Identifiers Ensembl protein databases using the provided accessory data. The resulting interaction data were filtered to contain only interactions with a high confidence STRING combined score (i.e. > 700) [57]. For network layout calculation, the combined score was used as an edge weight. The PPI network was constructed using Cytoscape software to visually represent the interactions among the potential targets identified in this study. Node size and color corresponded to their respective degree values, with larger and more vibrant nodes representing higher degrees. Edge thickness and darkness were proportionate to their connectivity scores, signifying that thicker and darker edges were indicative of higher connection scores [20].

### Plasmid construction

The full-length Lrg1 (Flag-tagged) plasmid, as well as extracellular domain plasmids for Alk1 (His-tagged), Tgfbr1/Alk5 (HA-tagged), Tgfb1 (Myc-tagged), Tgfbr2 (GST-tagged), and Eng (V5-tagged) were constructed by GeneChem. The HEK293T cells were transfected with Lipo8000 (Beyotime, C0533) according to the manufacturer’s protocol.

### Co-IP

As previously mentioned [53], cocultured primary microglial cells from wt mice were gently washed in PBS and treated with immunoprecipitation kit with protein A+G magnetic beads (Beyotime, P2179S). Simply put, cells were lysed in IP buffer containing protease inhibitor cocktail (Beyotime, P2179S-3) for 30 min and extraction was followed by centrifugation for 10 min at 12,000 rpm to obtain protein supernatant. The supernatant was preincubated with an appropriate amount of anti-Lrg1 (Santa, sc-390920, diluted 1:50) antibody or control IgG (Beyotime, P2179M-5) antibody (1 μg) at 4 °C overnight, followed by slow rotation and incubation with the A+G magnetic bead (Beyotime, P2179M-4) suspension at room temperature for 1 h. After incubation, the suspension was placed on a magnetic rack for 10 s to separate beads, washed with lysis buffer, and eluted by boiling in 1% sodium dodecyl sulfate (SDS) sample buffer for 10 min, then centrifuged at 12,000 rpm for 5 min, and the lysates were collected for SDS-polyacrylamide gel electrophoresis analysis and blotting.

According to the experimental plan, the HEK293T cells were transfected or cotransfected with single or multiple plasmids in serum-free medium (Yeasen, 40141ES80). After post-transfection 72 h, cells and serum-free media containing individual proteins or extracellular domains of TGF-β pathway receptors were collected separately, and manipulation of the former was performed according to the detailed steps described above, and relative medium containing the individual proteins or the extracellular domains of the TGF-β pathway receptors were incubated rotationally at 4 °C for immunoprecipitation. The primary antibody used for immunoprecipitation contained the following: Mouse anti-DYKDDDDK-tag monoclonal Antibody (CST, #8146); Mouse anti-His-tag monoclonal Antibody (MEDICAL & BIOLOGICAL LABORATORIES CO., LTD., MBL, D291-3); Mouse anti-HA-Tag(26D11) monoclonal Antibody (Abmart, M20003); Mouse anti-Myc-tag monoclonal Antibody (Proteintech, 60003-2-Ig); Mouse anti-GST-tag monoclonal Antibody (ABclonal, AE001); and Mouse anti-V5-tag monoclonal Antibody (ABclonal, AE017).

### Western blotting

Proteins were lysed from mouse brain tissue and cells using RIPA lysis buffer, or extracted from immunoprecipitation. Protein (20 to 30 μg) was loaded onto 8% to 12% SDS-polyacrylamide gels. Proteins were electrophoresed until fully separated, then transferred to a polyvinylidene fluoride membrane. The membrane was blocked at room temperature for 1 h with 5% skim milk or bovine serum albumin, and then incubated overnight at 4 °C with the following primary antibodies: Rabbit anti-Lrg1 polyclonal antibody (Proteintech, 13224-1-AP, diluted 1:100); Rabbit anti-Il-1b polyclonal antibody (Baijia Biotechnology Co., Ltd., IPB0002); Rabbit anti-interleukin 6 (Il-6) polyclonal antibody (Baijia Biotechnology Co., Ltd., IPB0062); Rabbit anti-interleukin 4 (Il-4) polyclonal antibody (Baijia Biotechnology Co., Ltd., IPB3904); Rabbit anti-interleukin 10 (Il-10) polyclonal antibody (Baijia Biotechnology Co., Ltd., IPB0104); Rabbit anti-Tgfb1 polyclonal antibody (ABclonal, A16640); Rabbit anti-nitric oxide synthase 2 (Nos2) polyclonal antibody (Proteintech, 22226-1-AP); Rabbit anti-glyceraldehyde-3-phosphate dehydrogenase (GAPDH) polyclonal antibody (Proteintech, 10494-1-AP); Rabbit anti-Alk1 polyclonal antibody (Huabio, HA722529); Rabbit anti-Alk5 polyclonal antibody (Immunoway, YM8858); Rabbit anti-Tgfbr2 polyclonal antibody (Immunoway, YM8220); Rabbit anti-Eng polyclonal antibody (Proteintech, 10862-1-AP); Rabbit anti-Mothers Against Decapentaplegic Homolog 1/5 (Smad1/5) polyclonal antibody (immunoway, YT4325); Rabbit anti-Smad2/3 polyclonal antibody (Wanleibio, wl01520); Rabbit anti-phospho-Smad1/5 (p-Smad1/5) polyclonal antibody (Huabio, HA722566); Rabbit anti-p-Smad2/3 polyclonal antibody (Wanleibio, WL02305); Rabbit anti-DYKDDDDK-tag monoclonal antibody (Proteintech, 20543-1-AP); Rabbit anti-His-tag polyclonal antibody (Proteintech, 10001-0-AP); Rabbit anti-HA-Tag(26D11) polyclonal antibody (Proteintech, 51064-2-AP); Rabbit anti-myelocytomatosis oncogene (Myc)-tag monoclonal antibody (Abcom, ab9106); Rabbit anti-glutathione S-transferase (GST)-tag monoclonal antibody (ABclonal, AE006); and Rabbit anti-V5-tag polyclonal antibody (Proteintech, 14440-1-AP). Membranes were washed 3 times by Tris-buffered saline with tween-20 (TBST) and incubated at room temperature for 1 h with horseradish peroxidase-conjugated secondary antibody (goat anti-rabbit IgG). Immunoreactivity was assessed using an enhanced chemiluminescence (ECL) kit (Baiuoleji [Hubei] Biotechnology Co., Ltd., BOLG001). Immunolabeling was scanned using a chemiluminescent imaging system, and data were normalized to GAPDH.

### Statistical analysis

Sequencing data were statistically analyzed using R Studio (version 4.2.0). Western blot results were analyzed using ImageJ (version 1.54) to quantify gray values. Statistical analysis was performed using GraphPad Prism software (version 9.5). The gray values of bands and fluorescence cell counts were shown as the mean ± standard deviation (SD) and analyzed using one-way analysis of variance (ANOVA) followed by Tukey’s post hoc tests for multiple comparisons. The model and functional scores of animal models were presented as median (interquartile range), analyzed using Kruskal–Wallis *H* with Dunn test. *P* value < 0.05 was considered statistically significant. *P < 0.05, **P < 0.01, ***P < 0.001 compared with controls.

## Ethical Approval

All animal experiments in this study were conducted in accordance with the ARRIVE 2.0 guidelines and the National Standard of the People’s Republic of China, Laboratory Animals - General requirements for animal experiment (GB/T 35823-2018), Guideline for ethical review of animal welfare (GB/T 35823-2018), and approved by the Laboratory Animal Ethics Committee of Renmin Hospital of Wuhan University (IACUC Issue No. WDRM20240702B). Animal experiments, outcome measurements, and data analysis were conducted by different personnel who were blinded to each other’s work. All possible efforts were exerted to minimize the suffering of animals and the quantity of animals utilized.

## Data Availability

All experimental and statistical data are available in the main text or the Supplementary Materials. Raw transcriptomics data can be obtained from the corresponding authors upon request, and access is restricted to noncommercial research consistent with applicable Chinese laws and regulations. Proper citation of this publication is required for any use of the data or information derived from this study.
