# Supplementary material for: Aerobic Exercise-Induced TGF-β Receptor Reprogramming Disrupts Neutrophil–Microglia Crosstalk to Attenuate Early Brain Injury after Subarachnoid Hemorrhage
Source: Research (Wash D C). 2026 May 28;9:1301. doi: 10.34133/research.1301 (PMC13216690; doi:10.34133/research.1301)
Supplement: Supplementary 1 — Table S1 Figs. S1 and S2 Data S1 to S19 [file research.1301.f1.zip › Data S1 Western blot.docx]

Fig. 1


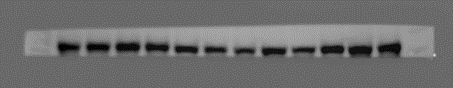

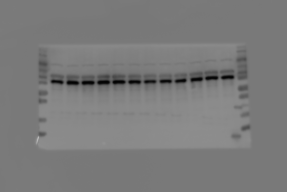
 GAPDH


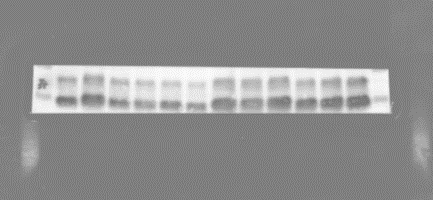

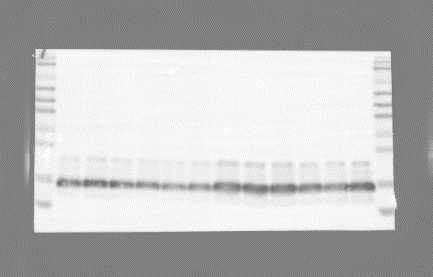
Il-1b


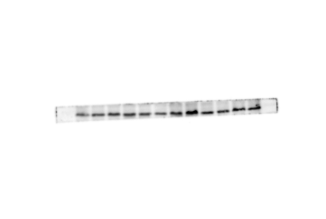

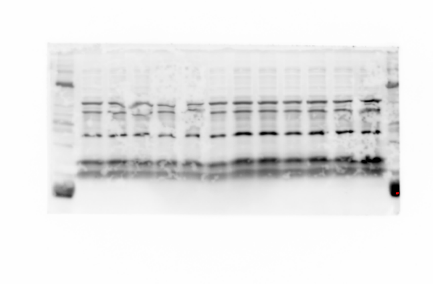
Il-6


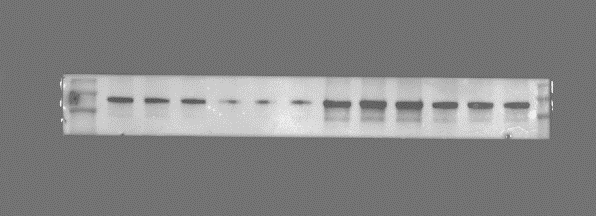

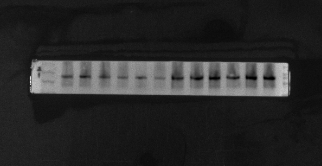
iNOS


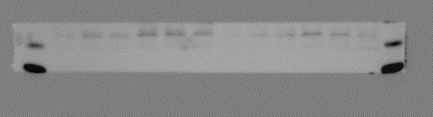

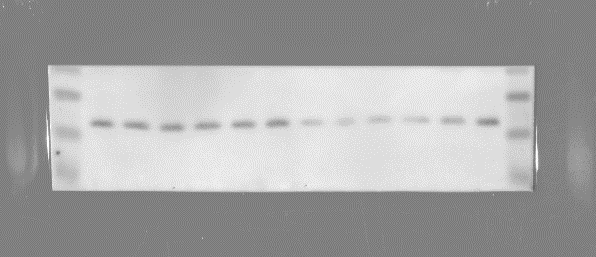
Il-4


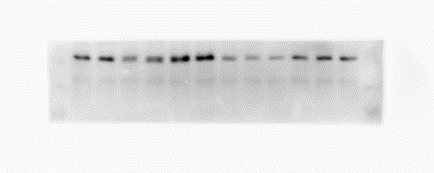

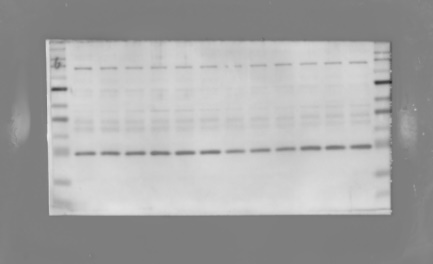
Il-10


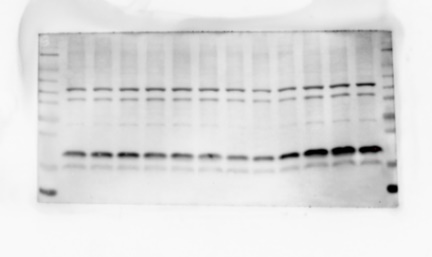
Tghfb1


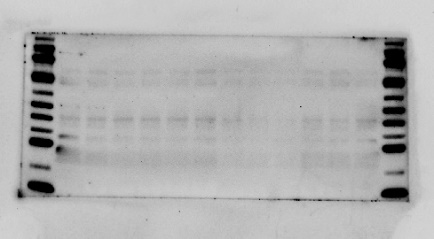


Fig. 2


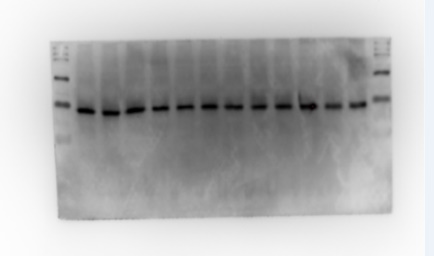

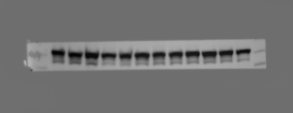
GAPDH


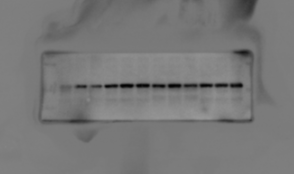

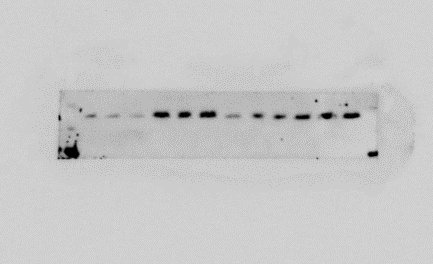
Il-1b


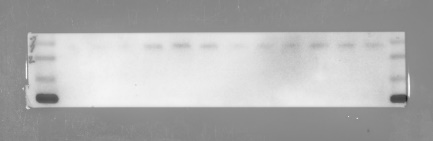

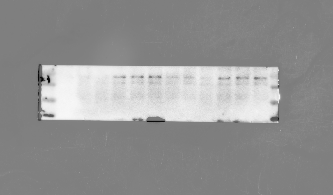
Il-6


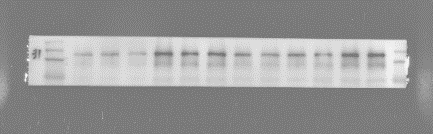

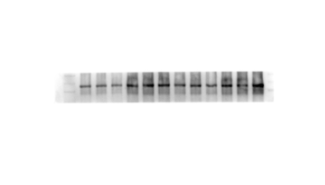
iNOS


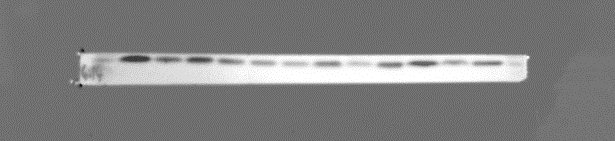

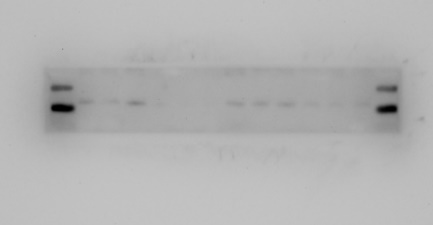
Il-4


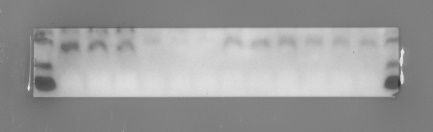

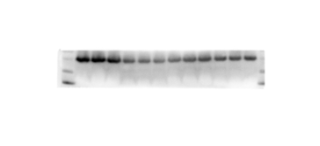
Il-10


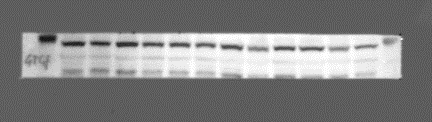

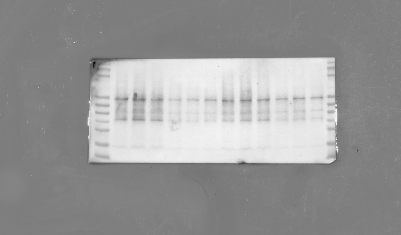
Tgfb1

Fig. S3


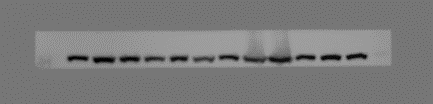

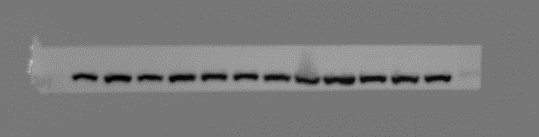
GAPDH


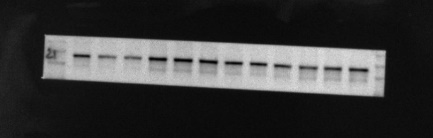

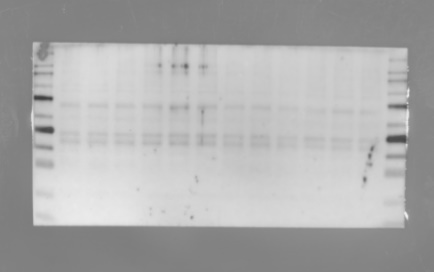
Alk1


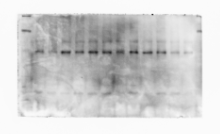

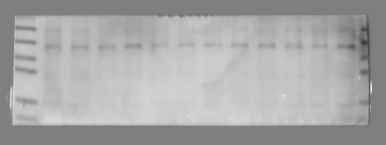
Smad1/5


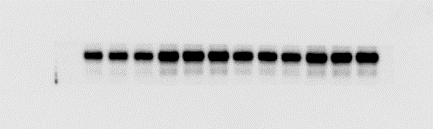

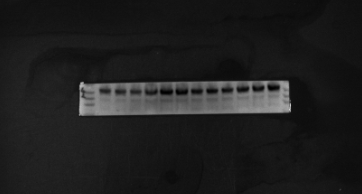
p-Smad1/5


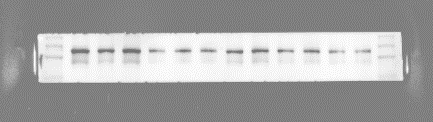

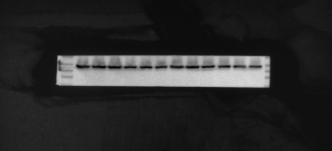
Alk5


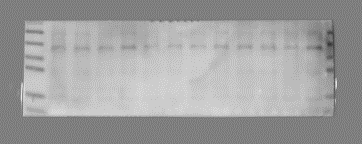

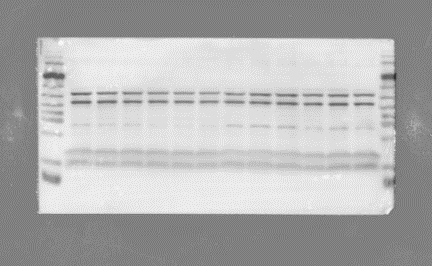
Smad2/3


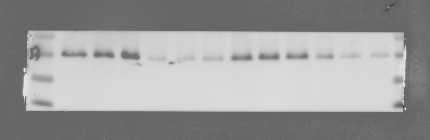

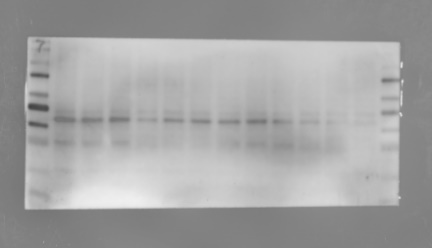
p-Smad2/3

Fig. 4


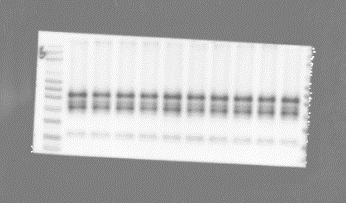

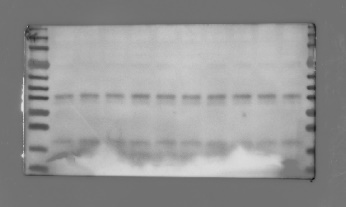

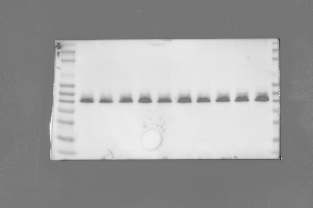
GAPDH


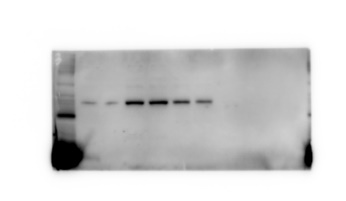

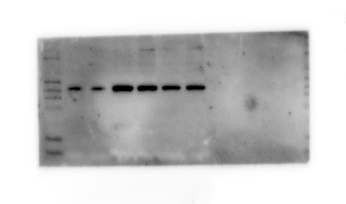

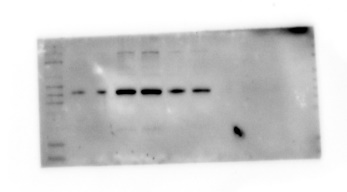
Lrg1


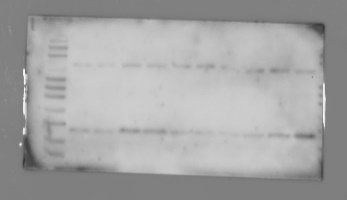

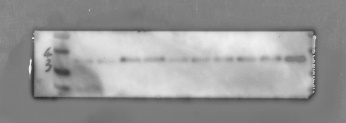

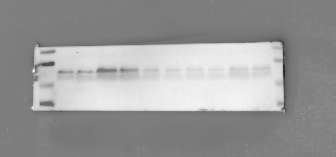
Il-1b


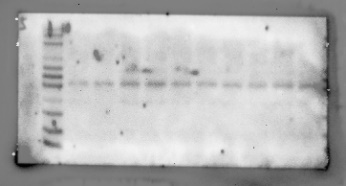

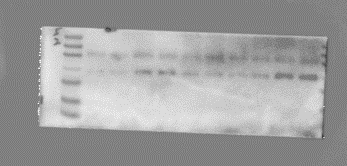

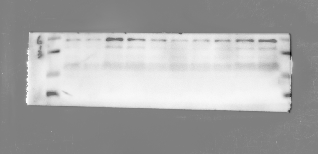
Il-6


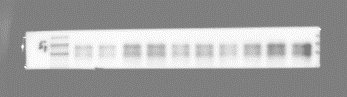

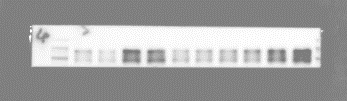

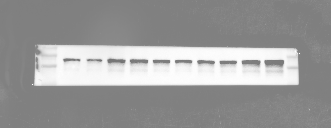
iNOS


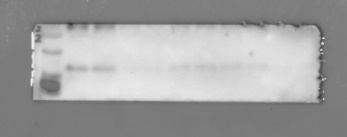

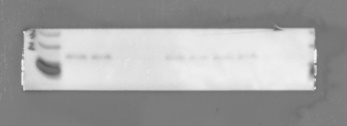

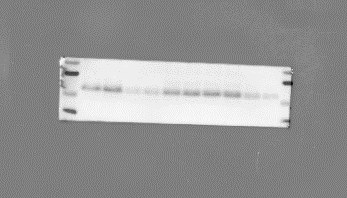
Il-4


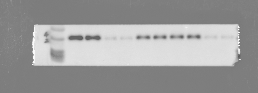

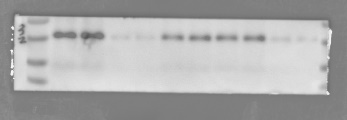

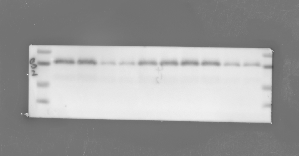
Il-10


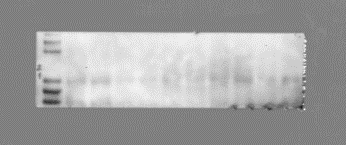

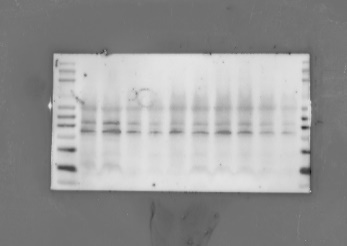

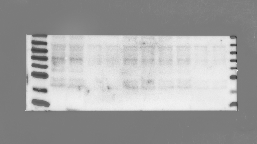
Tgfb1

Fig. 5


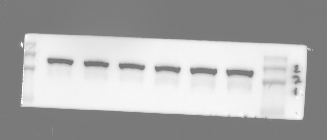

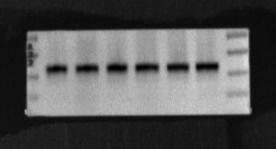

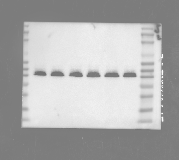
GAPDH


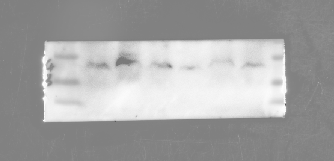

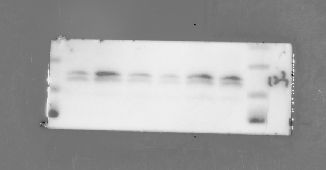

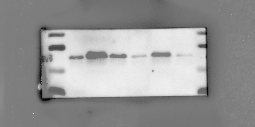
Il-1b


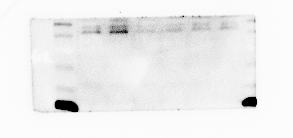

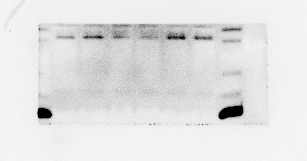

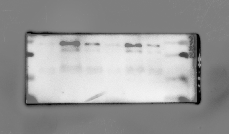
Il-6


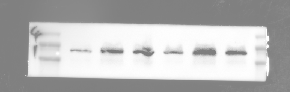

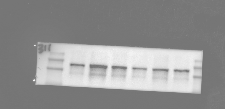

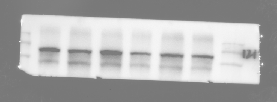
iNOS


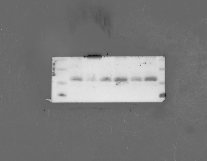

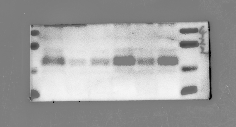

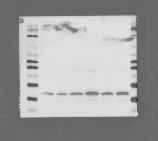
Il-4


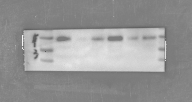

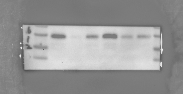

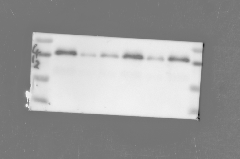
Il-10


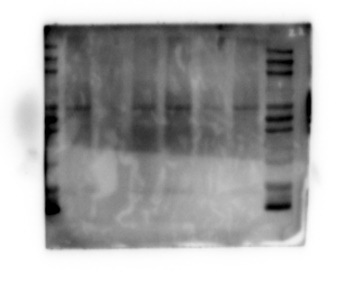

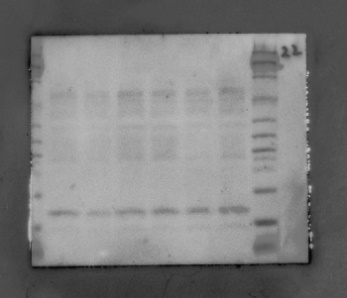

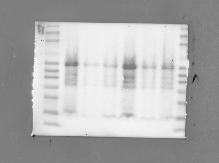
Tgfb1


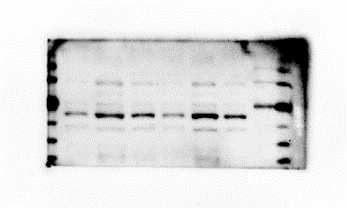

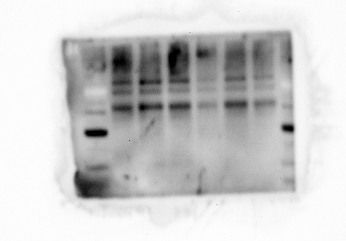

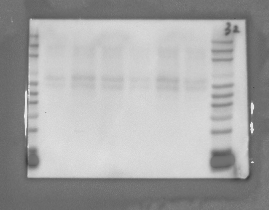
Alk1


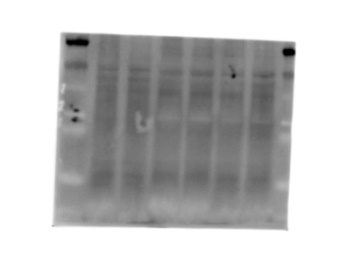

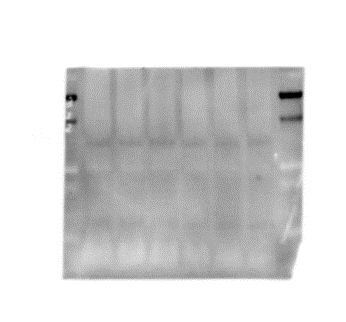

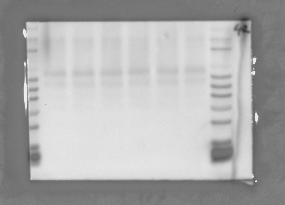
Smad1/5


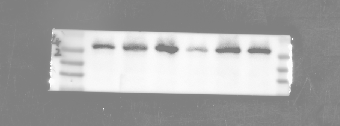

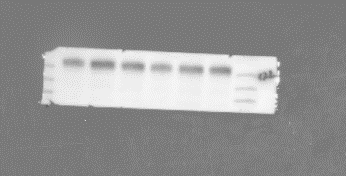

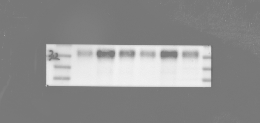
p-Smad1/5


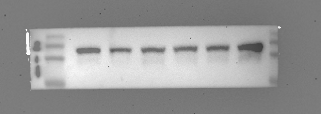

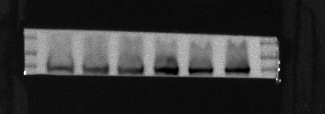

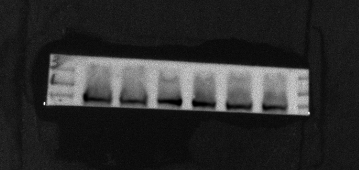
Alk5


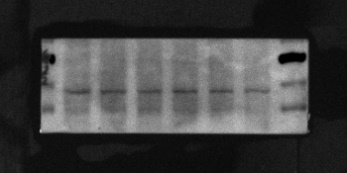
Smad2/3

p-Smad2/3
