# Supplementary material for: Aerobic Exercise-Induced TGF-β Receptor Reprogramming Disrupts Neutrophil–Microglia Crosstalk to Attenuate Early Brain Injury after Subarachnoid Hemorrhage
Source: Research (Wash D C). 2026 May 28;9:1301. doi: 10.34133/research.1301 (PMC13216690; doi:10.34133/research.1301)
Supplement: Supplementary 1 — Table S1 Figs. S1 and S2 Data S1 to S19 [file research.1301.f1.zip › Table S1 Fig. S1 to S2.docx]

Supplementary Materials for

**Aerobic exercise-induced TGF-β receptors reprogramming disrupts neutrophil-microglia crosstalk to attenuate early brain injury after subarachnoid hemorrhage**

Shengming Jiang, Li Jiang, Shiqiang Zhang et al.

*Mingchang Li. mingcli@whu.edu.cn

*Jianming Liao. ljm1992@whu.edu.cn

*Zhan Zhang doctorzhang2003@163.com

**This file includes:**

Table S1

Fig. S1 to S2

Data S1 to S19

Table S1.

| Groups | Mortality rate | Exclude |
| --- | --- | --- |
| **Experiment 1** |  |  |
| SE+Sham | 0% (0/24) | 0 |
| AE+Sham | 0% (0/24) | 0 |
| SE+SAH | 22.58% (7/31) | 3 |
| AE+SAH | 14.29% (4/28) | 2 |
| **Experiment 2** |  |  |
| SE+Sham | 0% (0/12) | 0 |
| SE+SAH | 29.41% (5/17) | 3 |
| AE+SAH | 14.29% (2/14) | 2 |
| AE+SAH+SB431542 | 25.00% (4/16) | 1 |
| **Experiment 3** |  |  |
| wt+SE+Sham | 0% (0/12) | 0 |
| wt+SE+SAH | 25.00% (4/16) | 2 |
| wt+AE+SAH | 14.29% (2/14) | 1 |
| ko+ SE+SAH | 20.00% (3/15) | 2 |
| ko+ SE+SAH+SB431542 | 25.00% (4/16) | 1 |
| Total |  |  |
| Sham | 0% (0/72) | 0 |
| SAH | 20.96% (35/167) | 17 |

Table S1. Grouping in animal experiments, and statistics on mortality rates and exclusion number. Mice with a total SAH score below 8 were excluded from the study.

Fig. S1


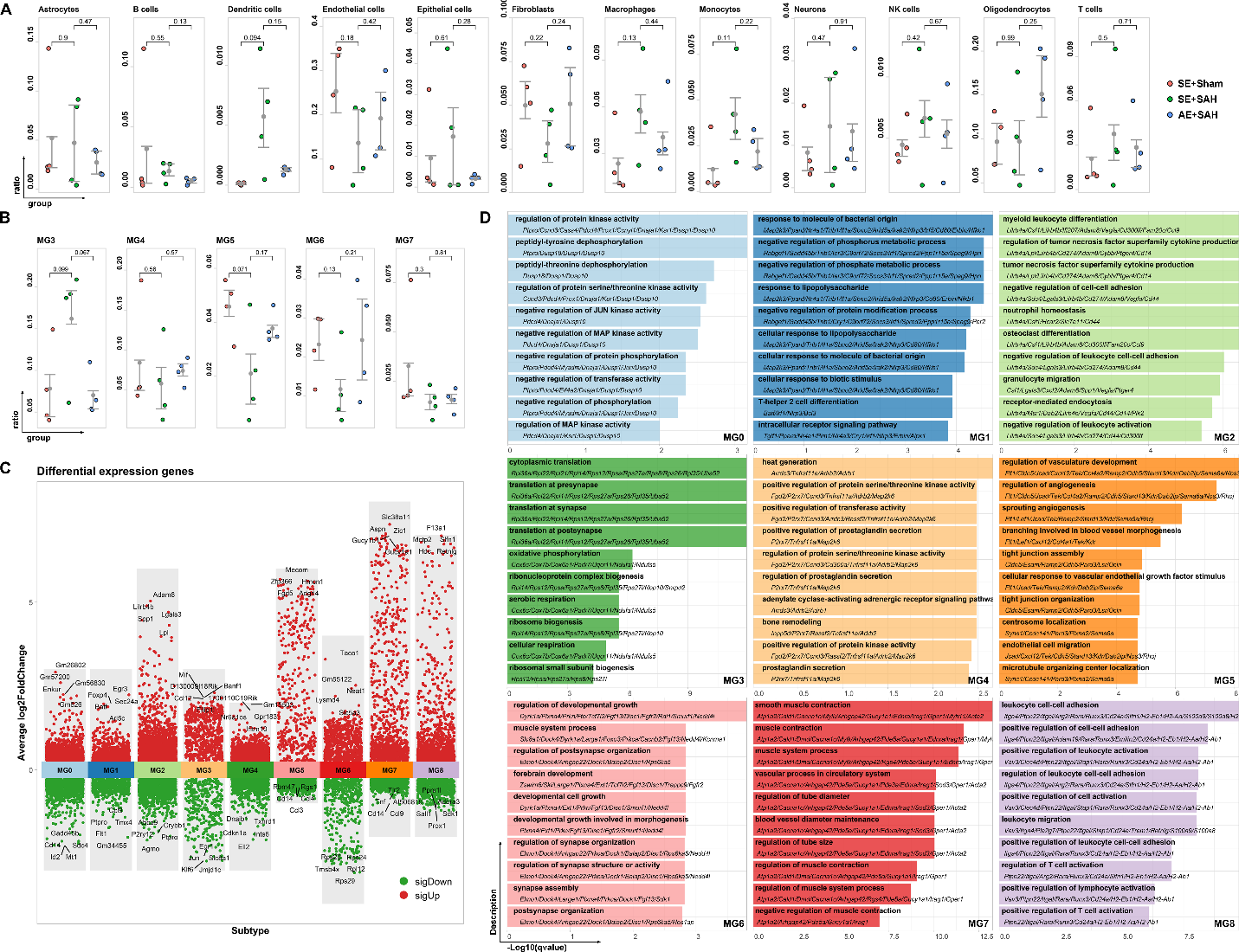


**Fig. S1** (**A**) Differences in the distribution of other clusters proportions between groups. (**B**) Differences in the distribution of other microglial subtypes proportions between groups. (**C**) Volcano plot of differentially expressed genes in microglial subtypes. (**D**) GO functional enrichment analysis of microglial subtypes.

Fig. S2


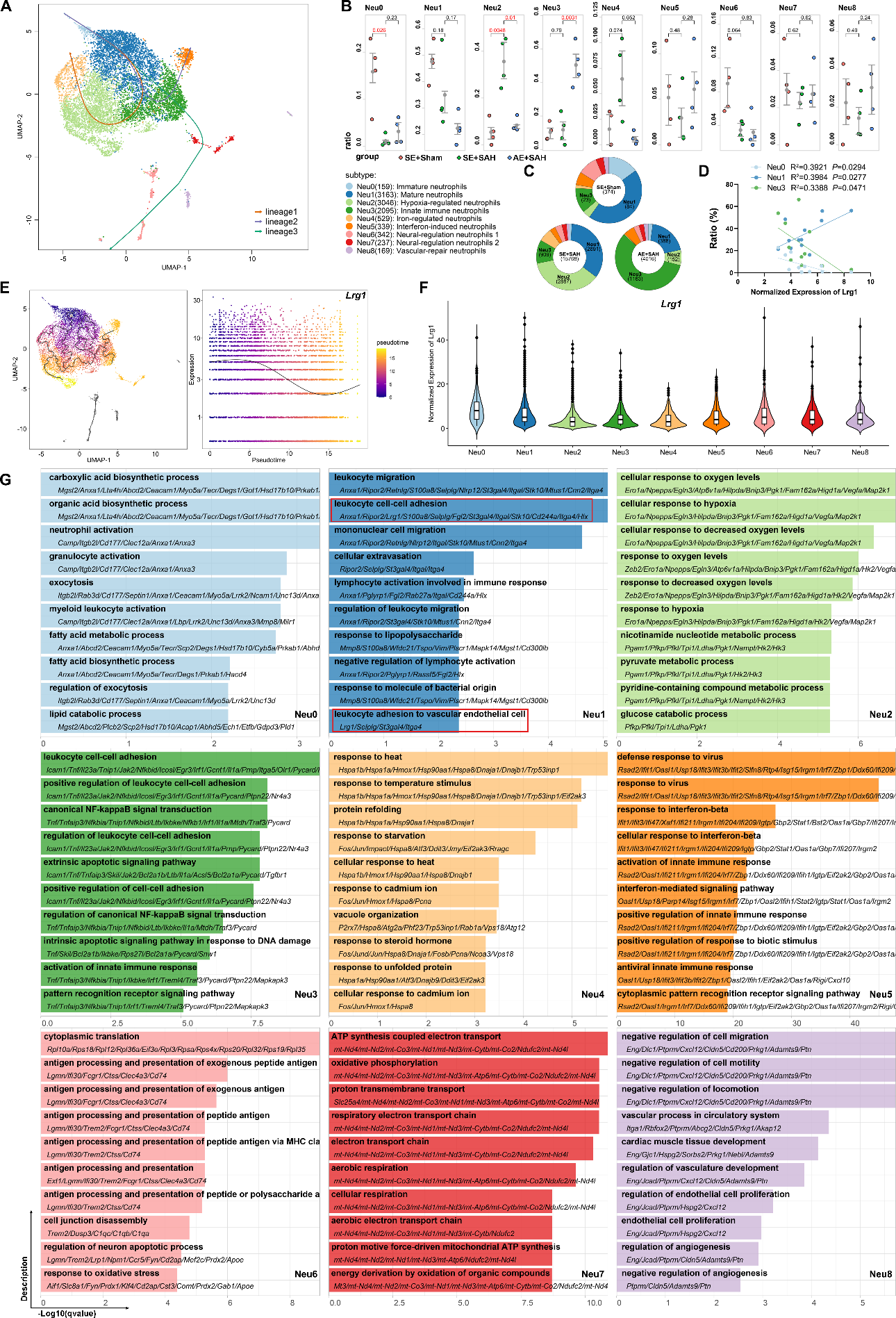


**Fig. S2** (**A**) The UMAP map of neutrophils. (**B**) Differences in the distribution of neutrophils subtypes proportions between groups. (**C**) The number of major subtypes of neutrophils in each group. (**D**) Correlation analysis and visualization of Lrg1 normalized expression with proportional distribution of major neutrophils subtypes with statistical differences. (**E**) The pseudotime time series calculation results of the Monocle3 software package based on umap clustering for neutrophil subtypes, and the pseudotime expression of the Lrg1 protein. (**F**) The normalized expression of Lrg1 in neutrophils subtypes. (**G**) GO functional enrichment analysis of neutrophils subtypes.
